# Mesenchymal stromal cell mitochondrial transfer to human induced T-regulatory cells mediates FOXP3 stability

**DOI:** 10.1038/s41598-021-90115-8

**Published:** 2021-05-21

**Authors:** Jeong-su Do, Daniel Zwick, Jonathan D. Kenyon, Fei Zhong, David Askew, Alex Y. Huang, Wouter Van’t Hof, Marcie Finney, Mary J. Laughlin

**Affiliations:** 1 Department of Immunology and Theranostics, Diabetes and Metabolism Research Institute, The Beckman Research Institute, City of Hope National Medical Center, 1500 E. Duarte Road, Duarte, CA 91010 USA; 2 Cleveland Cord Blood Center, 25001 Emery Rd, Cleveland, OH 44106 USA; 3 Department of Pediatrics, School of Medicine, Case Western Reserve University, Cleveland, OH 44106 USA; 4 Department of Biomedical Engineering, School of Medicine, Case Western Reserve University, Health Education Campus, 9501 Euclid Ave, Cleveland, OH 44106 USA

**Keywords:** Immunology, Autoimmunity, Immunotherapy

## Abstract

The key obstacle to clinical application of human inducible regulatory T cells (iTreg) as an adoptive cell therapy in autoimmune disorders is loss of FOXP3 expression in an inflammatory milieu. Here we report human iTreg co-cultured with bone marrow-derived mesenchymal stromal cells (MSCs) during short-term ex vivo expansion enhances the stability of iTreg FOXP3 expression and suppressive function in vitro and in vivo, and further that a key mechanism of action is MSC mitochondrial (mt) transfer via tunneling nanotubules (TNT). MSC mt transfer is driven by mitochondrial metabolic function (CD39/CD73 signaling) in proliferating iTreg and promotes iTreg expression of FOXP3 stabilizing factors BACH2 and SENP3. These results elucidate cellular and molecular mechanisms underlying human MSC mt transfer to proliferating cells. MSC mt transfer stabilizes FOXP3 expression in iTregs, thereby enhancing and sustaining their suppressive function in inflammatory conditions in vitro and in vivo.

## Introduction

Human and murine CD4 T cells can be efficiently differentiated ex vivo in the presence of TGF-β to generate inducible regulatory T cells (iTregs) with equivalent suppressive capacity as thymus-derived T regulatory cells (tTreg)^1, 2^. iTregs have potentially enormous clinical application in diverse inflammatory conditions, including autoimmune disorders and graft versus host disease^3^. However, the instability of FOXP3 expression in iTregs, resulting in loss of suppressive function in inflammatory conditions, has to date limited wide clinical application^4^.

A number of studies have shown tTreg cells prevent adverse inflammation in multiple disease settings including autoimmunity and graft vs. host disease^5, 6^. Although ex vivo protocols to expand tTregs have been developed, the limited number and anergic nature of tTregs, the risk of effector T cell contamination during expansion^7–9^, and the instability of tTreg phenotype and suppressor function upon transfer to an inflammatory environment^10, 11^, remain significant obstacles to clinical efficacy.

Treg suppressive function is tightly controlled by Foxp3 co-transcriptional proteins^12, 13^. BACH2, a Broad-complex-tramtrack-Bric-a-Brac and Cap’n’collar homology 2 bZip transcription factor, has been shown to stabilize Treg Foxp3 expression in recent murine studies^14, 15^. BACH2 has also been shown to regulate FOXP3 expression in human Umbilical Cord Blood (UCB) iTreg^16^. Further, murine studies identify SUMO specific protease 3 (SENP3) to be a critical regulator of Treg function by regulating the SUMOylation and nuclear localization of BACH2^17^.

Expansion of T cells in IL-2 based standard suspension cultures in vitro is associated with cell differentiation and senescence^18^. We observed previously, the addition of feeder layer MSCs provides improved cytokine driven expansion of early UCB CD34(+)/CD38(−)/HLA-DR(−) hematopoietic progenitors and inhibits differentiation and rates of apoptosis during short-term cytokine driven in vitro expansion^19^. Further, previous work has shown that MSC improve recovery of injured neuronal cells via mitochondrial transfer^20, 21^. MSC have been identified to favor iTreg formation^22^, yet the potential of MSC to promote iTreg functional stabilization has not been explored.

Our intent with this study was to improve upon the current standard in the field of Treg expansion for clinical use, e.g. short-term 3 week IL-2 based suspension culture conditions. We examined whether iTreg co-culture over a mono-layer of mesenchymal stromal cells (MSCs) could minimize iTreg cell differentiation and senescence and enhance iTreg number and suppressive function during in vitro expansion vs. standard IL-2 media suspension culture conditions^23, 24^.

## Results

### MSC co-culture renders iTreg stability

**Figure 1:**
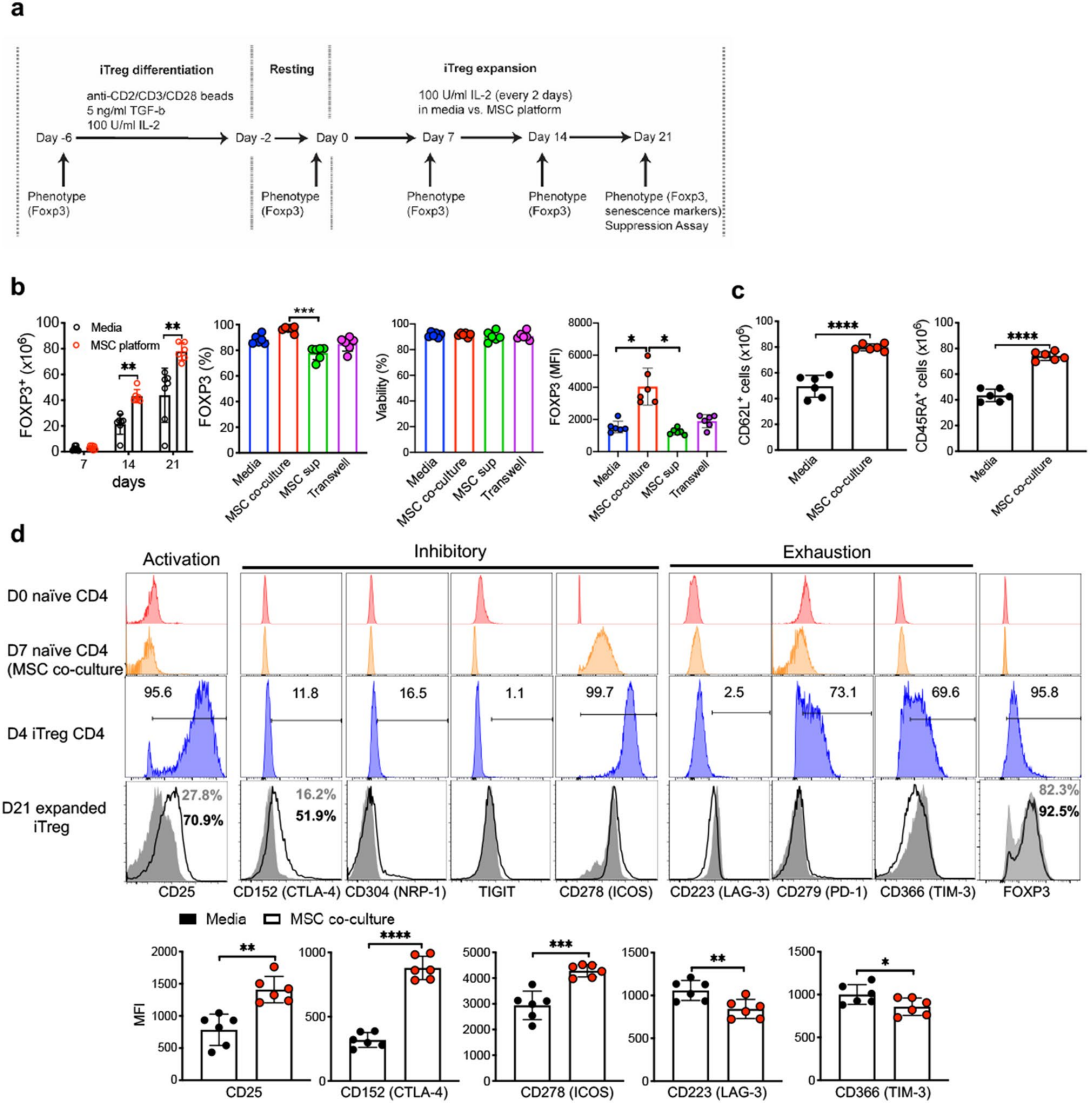
MSCs enhance and sustain intrinsic iTreg phenotype during IL-2 driven ex vivo expansion. (**a**) Schematic diagram shows experimental strategy for iTreg expansion. (**b**) Absolute number, percentage of FOXP3^+^, FOXP3 MFI in CD4 T cells and viability of CD4 T cells at day 21 were measured at indicated time points in IL-2/media vs MSC co-culture with identical IL-2/media (*n* = 6–7). Multiple comparisons analysis was performed using the Friedman test. (**c**) Absolute number of CD62L^+^ and CD45RA^+^ iTreg cells were calculated at 21 days expansion (*n* = 5–6). (**d**) Immuno-phenotyping of Treg marker expression on D0 naïve CD4 T cells, 7 days expanded naïve T cells in IL-2/media over MSC monolayer w/o CD3/CD28 stimulation (Day 7 naïve CD4—MSC co-culture), or 4 days stimulated CD4 T cells with TGF-β/IL-2 and CD3/28—(Day 4 iTreg CD4) and iTregs included surface staining with antibodies targeting CD25, CD152 (CTLA-4), CD223 (LAG-3), CD278 (ICOS) CD304 (NRP-1), CD279 (PD-1), CD366 (Tim-3), and TIGIT at 21 days expansion. Data are representative of three independent experiments ± SD. *p < 0.05, **p < 0.01, ***p < 0.001, ****p < 0.0001 paired t test. See also Supplementary Fig. S1.

Human marrow MSC exhibited morphology and phenotype (CD44, CD73, CD90 and CD105 expression) as previously described^25^ (Supplementary Fig. S1a). UCB-derived naive CD4^+^CD45RA^+^ T cells were differentiated into iTreg in standard 4 day culture in TGF-β condition^16^. UCB FOXP3^+^ iTregs co-cultured with MSCs rendered a significantly higher percentage and absolute number of FOXP3^+^ iTregs compared to standard IL-2/media suspension culture condition^26^ at day 14 and 21 and show similar viability (Fig. 1b and Supplementary Fig. 1b–d). We also examined CD62L and CD45RA, markers associated with Treg subsets with enhanced suppressive capacity^27, 28^ and naive phenotype^29^. CD62L^+^ and CD45RA^+^ iTregs were significantly increased during 21 day culture over MSCs (Fig. 1c and Supplementary Fig. 1e). Although prior studies point to paracrine secretion of factors by MSC as sufficient to enhance iTreg induction^30^, we did not observe enhanced FOXP3 expression via MSC-mediated paracrine effect in a trans-well system (Fig. 1b and Supplementary Fig. 1d). Intriguingly, iTregs co-cultured with MSC exhibited significantly higher FOXP3 expression (Supplementary Fig. 1d). We next investigated the impact of MSC feeder layer co-culture on expression of surface molecules involved in iTreg inhibitory function and any evidence of cell exhaustion. As shown in Fig. 1c, MSC co-culture was associated with significantly enhanced expression of several molecules on iTregs associated with suppressive T cell function, e.g. CD25, CD152, and CD278^31^. MSC platform iTregs also had significantly lower expression of T cell exhaustion surface markers (LAG-3, TIM-3) during short-term (21 day) culture in IL-2 (Fig. 1d). These observations suggest that MSC modulate iTreg phenotype and function during IL-2 mediated short-term expansion. Of note, expression of these iTreg markers was not observed in naive CD4 T cells on Day 0 prior to 4 day Treg induction (Fig. 1d). Robust expression of these markers was observed by Day 4 of iTreg differentiation, immediately prior to splitting into short-term expansion in IL-2/media suspension culture vs. identical IL-2/media on a monolayer of MSC feeder cells (Supplementary Fig. 1f,g). In further support of a role for MSC in Treg stabilization, we observed that MSC also enhance maintenance of Treg marker expression in tTregs isolated from UCB during 21 days of culture expansion over MSC (Supplementary Fig. 1g,h).

**Figure 2:**
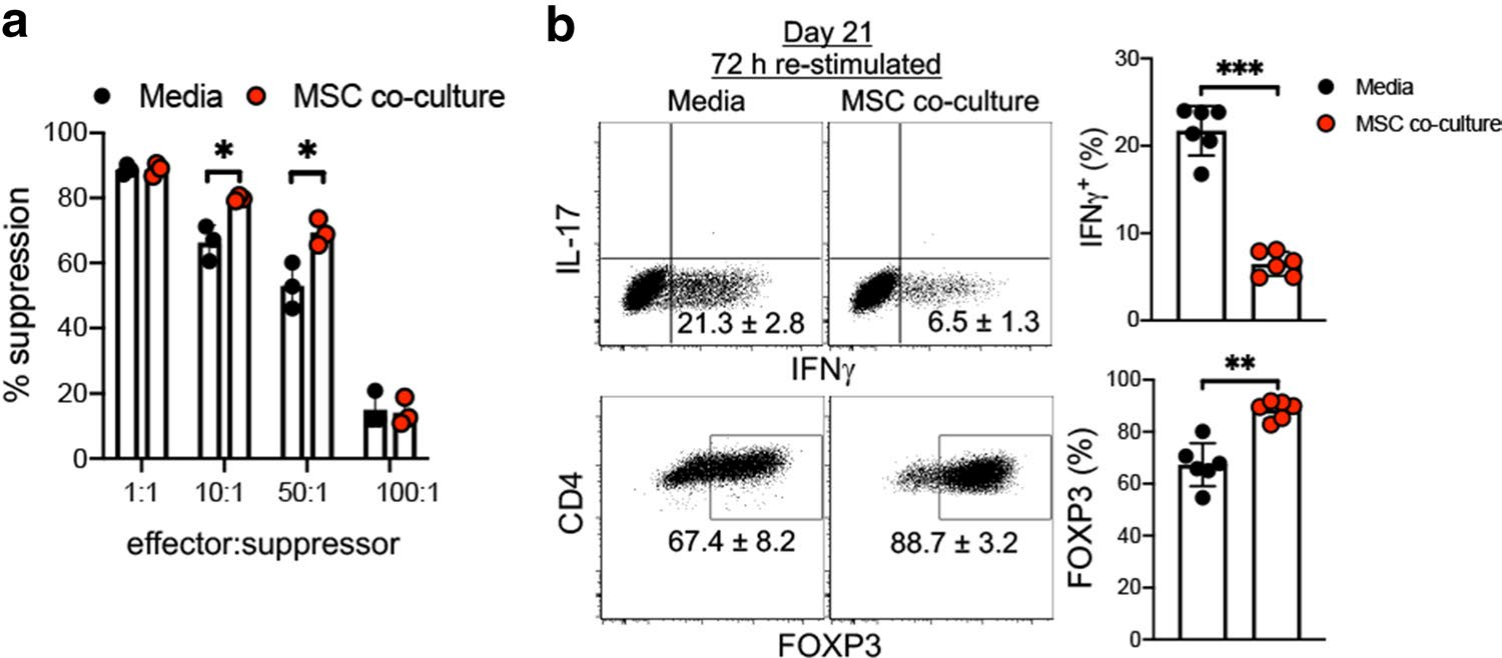
MSC co-culture stabilises FOXP3 expression in iTregs in the presence of inflammatory cytokines. (**a**) Suppressive effects of iTregs were measured day 21 of ex vivo expansion of iTregs in IL-2/media vs MSC co-culture, CD25^high^ FACS sorted iTregs were used to test suppressive function. 4 days after stimulation, CFSE dilution was examined by FACS (*n* = 3). (**b**) Expression of FOXP3 on iTregs was measured following 72 h restimulation with CD3 and CD28 antibodies (1 μg ml^−1^) in the presence of inflammatory cytokines IFNγ (10 μg ml^−1^), IL-6 (10 μg ml^−1^), and TNF (10 μg ml^−1^) at concentration of 5 × 10^5^ cells ml^−1^ and analyzed by FACS. Intracellular IFNγ and IL-17 were detected by flow cytometry analysis. Data are representative of two independent experiments ± SD (*n* = 6–7) **p < 0.01, ***p < 0.001 paired t test. See also Supplementary Fig. S2.

Next, we tested whether MSC co-culture enhances iTreg suppressive function^32^. MSC co-culture dramatically enhanced CD25^+^ isolated iTreg suppression of CD4 T cell proliferation in vitro (Fig. 2a and Supplementary Fig. 2a,b). A pronounced enhancement of iTreg suppression was observed even at very low 10:1 and 50:1 ratios compared with iTreg expanded in IL-2/media suspension culture conditions.

Inflammatory conditions destabilize iTreg phenotype and downregulate suppressive function^33–35^. We re-stimulated iTregs in the presence of pro-inflammatory cytokines to mimic an inflammatory environment and measured FOXP3 stability. MSC co-culture stabilized iTregs in this in vitro inflammatory environment (Fig. 2b). FOXP3 expression in MSC co-culture expanded iTregs remained > 90% when in resting state (n = 3, data not shown) in further support of enhanced iTreg stability. These data suggest that MSC feeder cells preserve iTreg FOXP3 expression and suppressive function during IL-2 driven short-term (21 day) expansion.

### MSC mt transfer requires direct cell–cell contact

**Figure 3:**
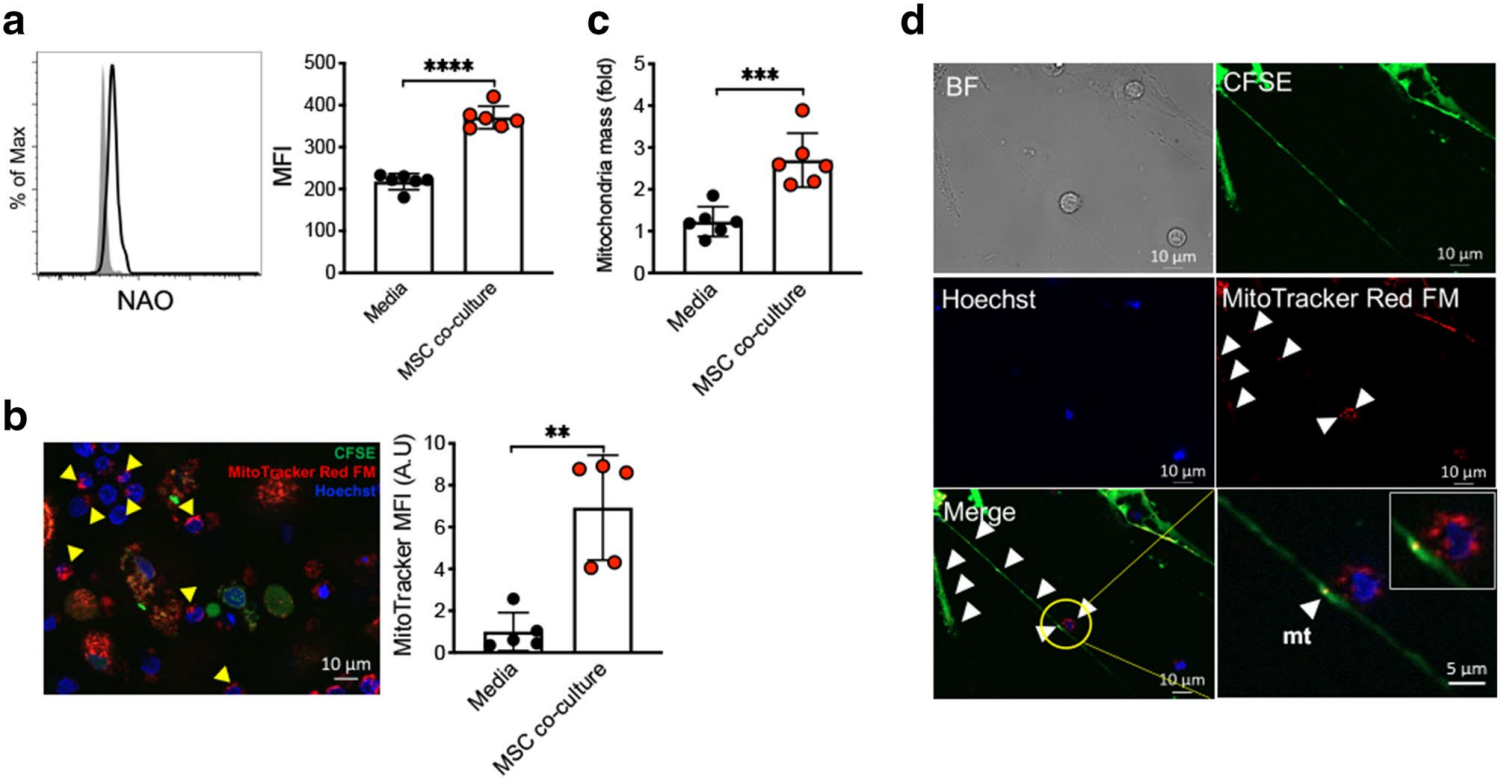
MSC mitochondrial transfer to iTregs occurs via TNT. (**a**) Mitochondrial quantity assay of iTregs was analyzed. At 3 weeks, media vs MSC co-culture iTreg cells were stained with NAO to quantify mt mass (*n* = 5). (**b**) Confocal imaging analysis for MitoTracker transfer. MSC were stained with CFSE (green) and MitoTracker Far Red (red), iTreg were stained with Hoechst (blue) and then co-cultured for 24–36 h (*n* = 5). (**c**) Mitochondria DNA copy number was assessed from IL-2/media or MSC co-culture expanded iTregs at day 21 (*n* = 6). (**d**) MSC mt transfer analysis. MSC were stained with CFSE (green) and MitoTracker Far Red (red), iTreg were stained Hoechst (blue) and cells were co-cultured for 24–36 h. Live cell images were collected by confocal microscopy. Data are representative of three independent experiments. (**b**,**d**) Analysis of recorded images was performed using Zen software Blue edition, (2011; version 2.0.14283.302). https://www.zeiss.com/microscopy/us/products/microscope-software/zen.html **p < 0.01, ***p < 0.001, ****p < 0.0001 paired t test. Scale bars: 10 μm. See also Supplementary Fig. S3.

**Figure 4:**
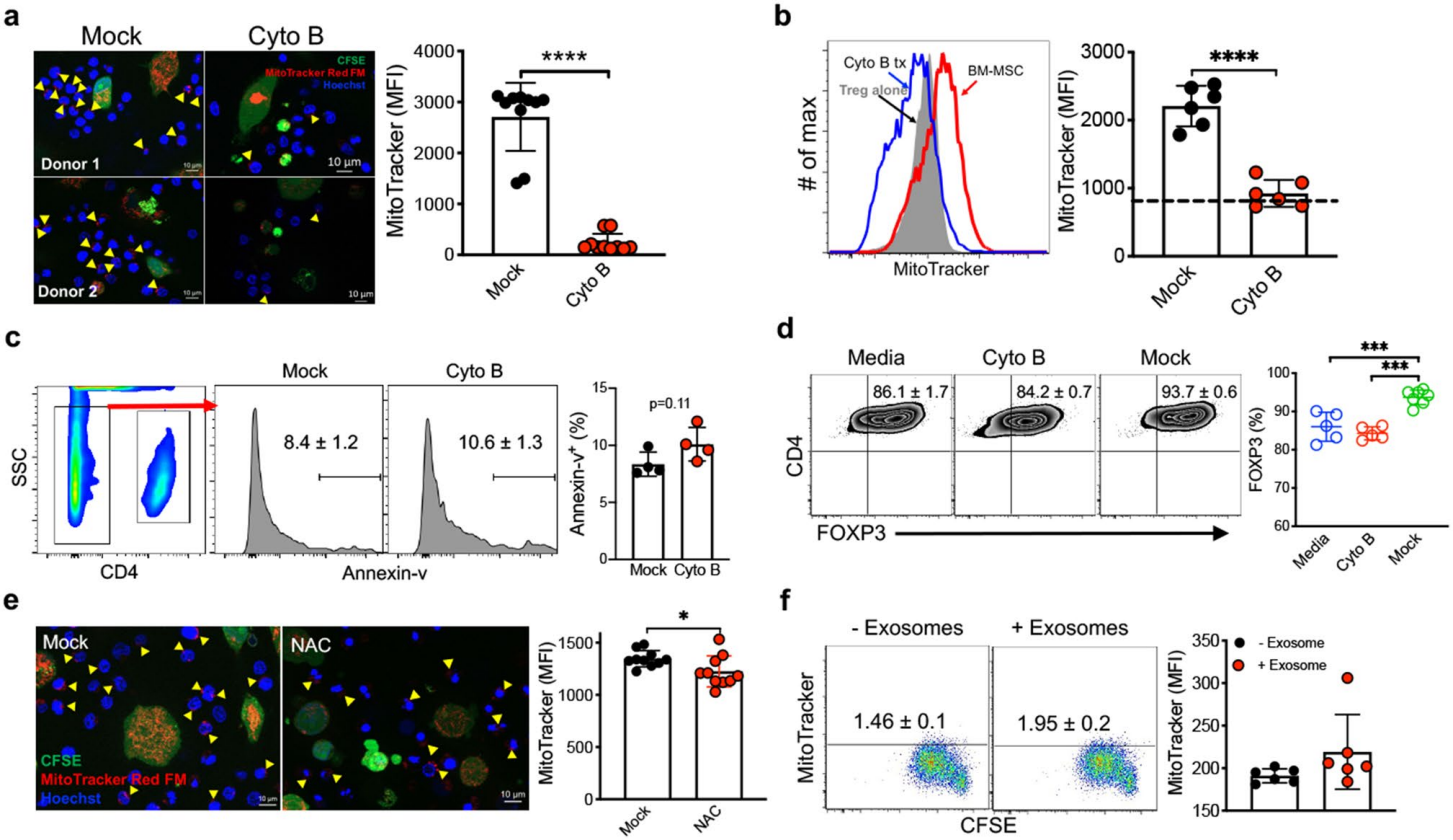
MSC-iTreg interactions are required for mt transfer. (**a**) Mitochondrial transfer measured in iTreg co-cultured with cytochalasin B treated MSC for 24–36 h (*n* = 5). Cytochalasin B was added in the MSC co-culture (350 nM). Arrowheads indicate mt transfer to iTreg. Analysis of recorded images was performed using Zen 2012 software. (**b**) FACS analysis of MSC mt transfer to iTreg after cytochalasin B treatment. MSC were stained with MitoTracker Far Red (red) and iTreg were co-cultured for 48–72 h. iTregs were stained by CD4 antibody and analyzed. (**c**) Annexin V FACS analysis of MSC viability after cytochalasin B treatment. MSCs were gated as CD4 negative population. (**d**) FOXP3 expression in iTregs was measured from mock vs cytochalasin B treated MSC co-culture expanded iTregs (*n* = 5–7). (**e**) Effect of ROS inhibitor treatment on mitochondrial transfer. MSC were pre-stained with CFSE and MitoTracker Red FM and then cultured with Hoechst stained iTreg for 24–36 h. The ROS inhibitor antioxidant *N*-acetylcysteine (NAC) 200 μM was added to MSC + iTreg culture for 24–36 h. Live cell images were collected by confocal microscopy. (**a**,**e**) Analysis of recorded images was performed using Zen software Blue edition, (2011; version 2.0.14283.302). https://www.zeiss.com/microscopy/us/products/microscope-software/zen.html (**f**) MSC exosome uptake by proliferating iTregs. MSC exosome were stained with MitoTracker Far Red (Hough et al., 2018). FOXP3^+^ iTregs were stimulated with plate bound CD3 (1 μg ml^−1^) for 72 h. Data are representative of 2–3 independent experiments. *p < 0.05, ***p < 0.001, ****p < 0.0001 paired t test. Scale bars: 10 μm. See also Supplementary Figs. S4 and S5.

Intercellular mt transfer by MSC has been previously described in neuronal injury and cancer models^36^. We measured total mt content in iTreg cells following MSC co-culture by flow cytometric analysis of iTregs labeled with the cardiolipin-binding dye nonyl acridine orange (NAO)^37^. MSC co-culture induced a ~ twofold increase in NAO mean fluorescence intensity in iTregs (Fig. 3a). To determine whether this resulted from increased endogenous iTreg mt biogenesis or MSC mt transfer, we labeled MSC mt with MitoTracker and enumerated MitoTracker positive iTreg in MSC co-cultures by direct visualization using epifluorescence microscopy. Intriguingly, robust mt transfer was observed (Fig. 3b), as the proportion of MitoTracker^+^ iTregs reached ~ 16% within 16 h MSC co-culture (Supplementary Fig. 3a). Expanded iTreg mt copy number was further increased in iTregs during short-term (21 day) expansion in MSC co-culture condition (Fig. 3c). Mitochondrial transfer was directly visualized via tunneling nanotubules (TNT) between MSCs and iTregs (Fig. 3d and Supplementary Fig. 3b,c). To determine whether MSC mt transfer was reliant on TNT for transfer to proliferating iTreg during IL-2 driven expansion culture, Cytochalasin B (Cyto B), an inhibitor of TNT formation, was added and completely blocked the transfer of MitoTracker^+^ mt from MSC to proliferating Tregs (Fig. 4a,b and Supplementary Fig. 3d). Of note, Cytochalasin B was utilized at a concentration (350 nM) known to inhibit TNT formation with minimal effects on cell viability in a range of cells including MSC, acute myeloid leukemia cells, and PC12 cells^38, 39^. Nevertheless, one possibility is that reduced mt transfer was mediated by cyto B induced cytotoxicity in MSCs. Thus, we measured apoptosis by Annexin V (AV) staining. No differences in the proportion of AV^+^ MSCs (Fig. 4c), or in AV + Tregs (Supplementary Fig. 3e) between mock and cyto B treatment conditions was observed. Further, pre-treatment of MSCs, but not iTregs, with cyto B inhibited mt transfer, indicating MSC mt transfer occurs via TNT formation from MSCs (Supplementary Fig. 3f,g). We next tested the impact of MSC mt transfer on FOXP3 stability in iTregs. Cytochalasin B treatment significantly reduced FOXP3 expression in iTregs expanded on MSC (Fig. 4d).

Cellular reactive oxygen species (ROS) are implicated as regulators of MSC mt transfer in cancer^39^. We incorporated treatment with the ROS scavenger NAC (*N*-acetyl-l-cysteine) and observed an incomplete block of MSC mt transfer (Fig. 4e). As mt transfer can be induced by MSC derived soluble and exosome-mediated factors, we tested MSC mt transfer via exosomes. MitoTracker^+^ mt were not detected in proliferating FOXP3^+^ iTregs in IL2/media in the presence of MSC exosomes (Fig. 4f). Further, using a transwell separating MSCs from iTregs, MitoTracker^+^ iTregs were not observed, suggesting direct cell–cell contact is necessary for efficient mt transfer (Supplementary Fig. 3d). In addition, MSC mt transfer to iTregs did not occur in quiescent iTreg and is dependent on iTreg proliferation driven by IL-2 (Supplementary Fig. 4a,b). It has been shown other mesenchymal like cells and fibroblasts also transfer mitochondria^40^. However, we did not observe mitochondrial transfer to iTregs from other cell types including CD133^+^ hematopoietic stem cells (HSC) or HUVEC cells (Supplementary Fig. 5a). Further, we observed MSCs did not transfer mitochondria to iTregs after induction of MSC mitochondrial dysfunction^40^ (Supplementary Fig. 5b). Taken together, these data support a key role for the transfer of mitochondria from MSC to proliferating iTreg in enhancing iTreg number and suppressive function during IL-2 driven ex vivo expansion.

### CD39/CD73 signaling plays a critical role in MSC mt transfer and stabilization of iTreg suppressive function

**Figure 5:**
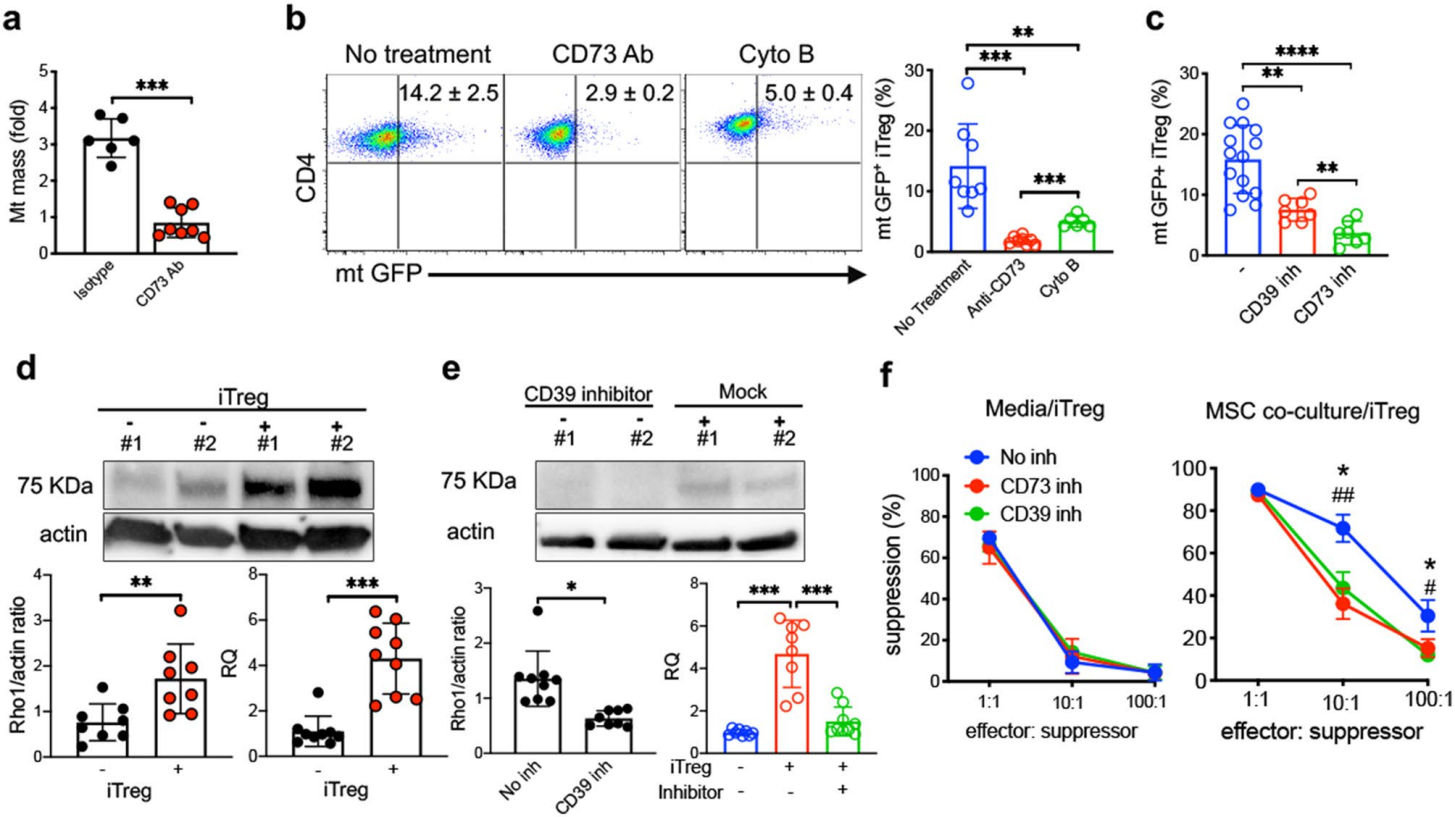
CD39/73 signaling induces MSC mitochondrial transfer to iTregs during IL-2 driven expansion. (**a**) Mitochondrial quantity was measured from IL-2/media or MSC co-culture expanded iTregs by RT-PCR (*n* = 6–8). Anti-CD73 antibody (10 μg ml^−1^) was added in MSC co-culture. Cells were collected at 72 h after mtGFP lentiviral transduced MSC co-culture. (**b**) Flow analysis of mtGFP^+^ iTregs in anti-CD73 blocking and cytochalasin B treatment. Cells were stained with CD4 antibody in order to identify iTregs at 72 h. (**c**) Analysis of mtGFP^+^ CD4^+^ iTregs after incubation with CD39 (100 nM) and CD73 (100 nM) inhibitor treated MSC (*n* = 7–14). Representative flow plot, gated for CD4^+^ iTreg cells after MSC co-culture. (**d**) Protein and RNA expression of Miro1 in MSC were measured after co-culture with iTreg. (**e**) After co-culture in the presence of CD39 inhibitor (*n* = 8–9). The image intensities for western blots were normalized to beta actin. Data are representative of 3–4 different experiments. (**f**) Effect of CD39 and CD73 inhibitors on suppressive functions of iTreg during MSC co-culture expansion. Inhibitor was added to iTreg and MSC co-culture. Data are representative of *n* = 3 independent samples. *p < 0.05, **p < 0.01, ***p < 0.001, ****p < 0.0001 paired t test. See also Supplementary Fig. S6.

Experiments were conducted to determine the mechanisms driving MSC mt transfer into proliferating iTregs. Treg express apyrases (CD39) and ecto-5'-nucleotidase (CD73) that contribute to inhibitory function by generating adenosine^41, 42^. Additionally, CD73-generated adenosine induces cortical actin polymerization via adenosine A1 receptor (A1R) induction of a Rho GTPase CDC42-dependent conformational change of the actin-related proteins 2 and 3 (ARP2/3) actin polymerization complex member N-WASP^43^. This is noteworthy since we identified MSC mt transfer to UCB iTreg occurs via cytochalasin B-sensitive, actin-dependent transport via TNTs. To test whether CD73 contributes to MSC mt transfer, we added CD73 blocking Ab to MSC co-cultures and measured iTreg mt mass. mt mass was significantly reduced in proliferating iTregs after CD73 blocking Ab treatment (Fig. 5a). To confirm MSC transfer mitochondria to iTregs during ex vivo expansion, we generated MSC stably expressing mitochondrially targeted GFP. MSCs were transduced with mito-GFP lentivirus to generate stable mtGFP^+^ MSCs (Supplementary Fig. 6a) and co-cultured with iTregs expanded in IL-2/media. Significantly less mtGFP^+^ iTregs were detected during 21 day co-culture with mito-GFP lentivirus transduced MSCs when CD73 signaling was blocked or TNT formation was inhibited (Fig. 5b and Supplementary Fig. 6b,c). As CD39 and CD73 calibrate purinergic signals delivered to immune cells through the conversion of ADP/ATP to AMP and AMP to adenosine, respectively^44, 45^, we inhibited each pathway and observed reduced mtGFP^+^ iTregs (Fig. 5c and Supplementary Fig. 6d).

As Miro1 has been shown to be a key regulator in mitochondrial intracellular transport^46^, we sought to determine if Miro1 played a role in MSC mt transfer to proliferating iTreg via TNT. We measured Miro1 expression in MSC after co-culture with iTregs. Co-cultivation with proliferating iTreg was associated with significantly enhanced expression of Miro1 in MSC, including both protein and RNA levels (Fig. 5d). Further, the enhanced MSC expression of Miro1 was inhibited by CD39 inhibition (Fig. 5e).

Since CD39/CD73 signaling play significant roles in mitochondrial transfer, we hypothesized these signaling pathways are critical to MSC-mediated enhancement of iTreg suppressive function. Inhibition of MSC mt into iTregs resulted in significantly reduced suppressive function vs. control (Fig. 5f, Supplementary Fig. 6d). These data indicate MSC enhance and stabilize iTreg FOXP3 expression and iTreg suppressive function via CD39/CD73-induced mitochondrial transfer to proliferating iTregs during IL-2 driven expansion.

### MSC mt transfer augments iTreg expression of FOXP3 stabilizing factors BACH2 and SENP3

**Figure 6:**
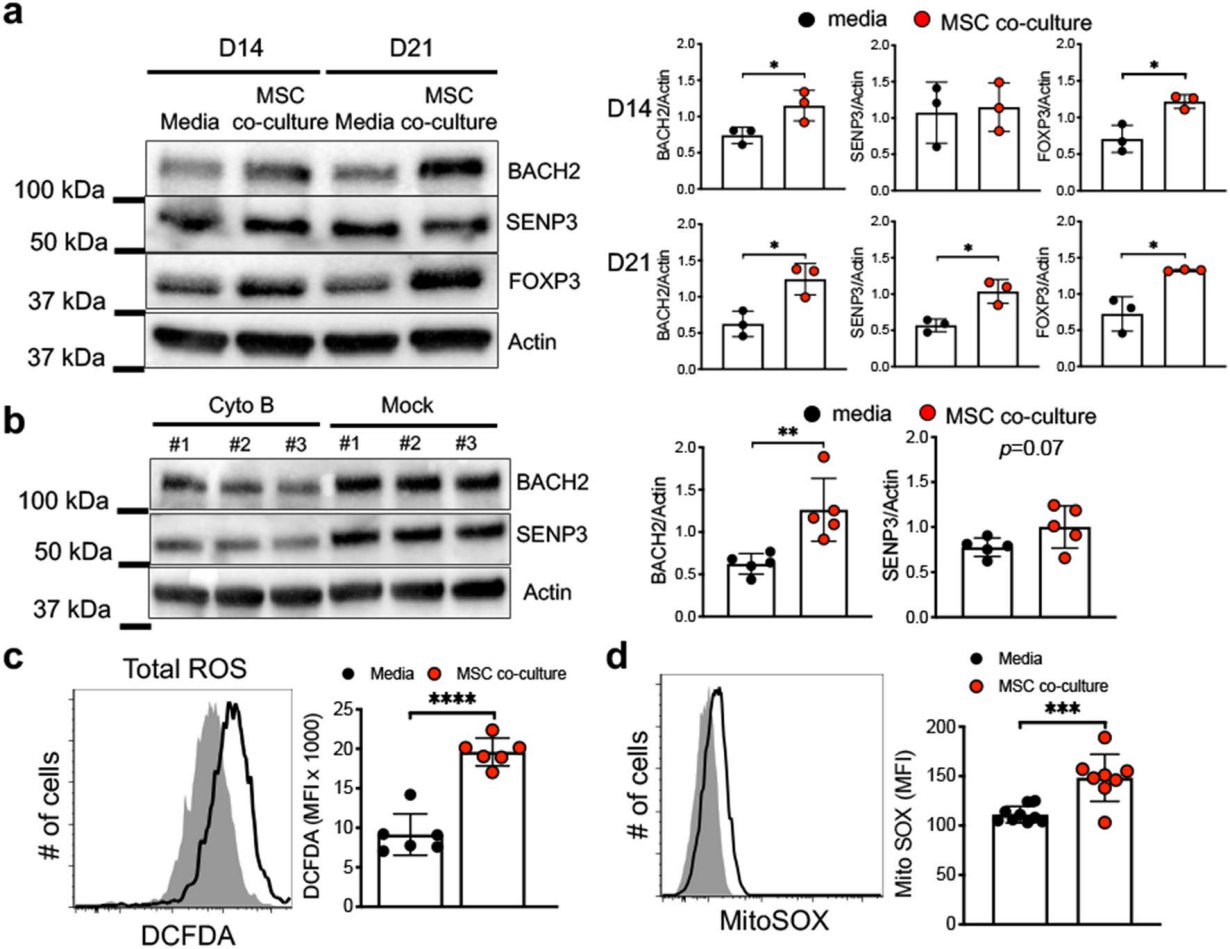
MSC co-culture induces iTreg BACH2 and SENP3 via ROS. (**a**) Expression of BACH2, SENP3, and FOXP3 on iTregs was measured by immunoblot assay. iTregs were expanded in media vs MSC co-culture and protein was isolated at indicated time. Data are representative of *n* = 3 independent samples. (**b**) Analysis of BACH2 and SENP3 on iTregs after CD39 signaling inhibition. iTregs were expanded in media vs MSC co-culture and protein was isolated at indicated time. Expression was measured by immunoblot assay. (**c**,**d**) Expression of intracellular reactive oxygen species (ROS) was measured by flow cytometry in media vs MSC co-culture expanded iTregs (**c**) expression of mitochondrial ROS was measured by FACS analysis (**d**) at 21 days expansion. Data are representative of *n* = 6 independent experiments. *p < 0.05, **p < 0.01, ***p < 0.001, ****p < 0.0001 paired t test. See also Supplementary Fig. S7.

BACH2 maintains stability and function of murine Treg^15, 47^. We previously identified BACH2 is highly expressed in human UCB-derived iTreg and increases FOXP3 expression^16^. Additional studies have shown that SENP3 modulates BACH2 SUMOylation of and enhances iTreg stability in response to changing environmental conditions, particularly intracellular ROS^17^. We postulated mt transfer from MSC modulates BACH2/SENP3 expression and/or function, given our observations MSC mt transfer exerts a positive effect on iTreg Foxp3 stability and suppressive function^17, 48, 49^. We measured iTreg BACH2 and SENP3 protein expression, comparing MSC co-culture and standard IL-2/media suspension conditions during short-term (day 14 and 21) in vitro expansion. iTreg FOXP3, BACH2 and SENP3 expression was significantly increased in MSC co-culture conditions (Fig. 6a). We next examined iTreg BACH2 and SENP3 protein levels after cytochalasin B treatment to block mt transfer and observed loss of BACH2 protein expression (Fig. 6b). Cytochalasin B treatment had no significant effect on iTreg SENP3 protein expression, as SENP3 activation and/or function may be altered in this setting (Fig. 6b).

Previous studies have shown that mtROS induces BACH2 expression in B cells^49^. We measured mtROS expression in iTregs and found MSC co-culture induced higher expression of total and mtROS compared to standard IL-2/media suspension culture conditions (Fig. 6c,d). To investigate whether mtROS is a critical factor for induction of BACH2 and SENP3, we utilized a ROS inhibitor in iTreg/MSC co-cultures. Notably, iTreg expression of BACH2 and SENP3 in MSC co-cultures were significantly reduced in the presence of the ROS inhibitor (Supplementary Fig. 7). Overall, these data are consistent with MSC mt transfer enhancing iTreg BACH2 expression and subsequent enhanced FOXP3 expression and stability.

### iTregs expanded on MSC feeder cells suppress effector CD4 and CD8 T cell responses in a xenogeneic GVHD NSG murine model

**Figure 7:**
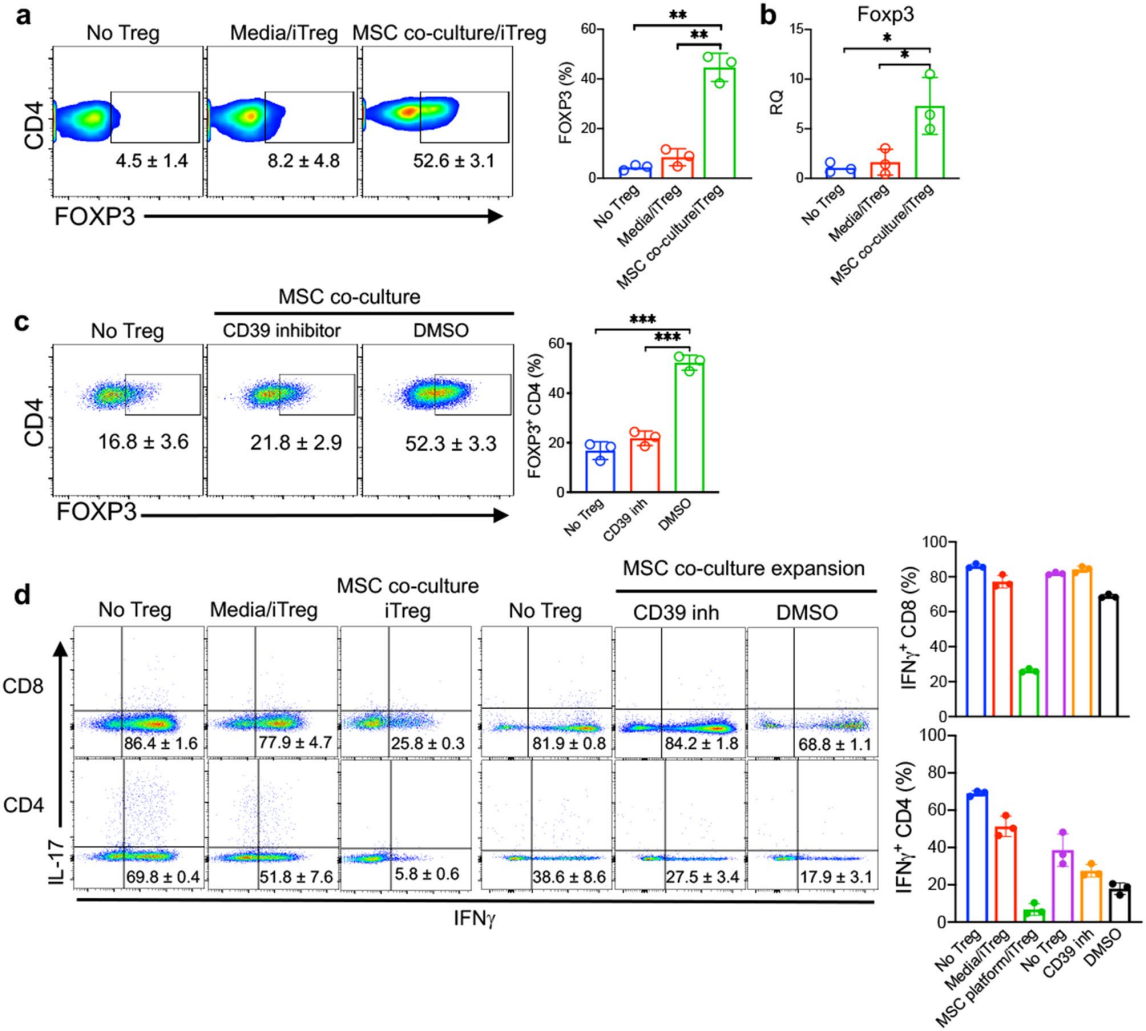
MSC co-culture expanded iTregs suppress effector T cell response in GVHD inflammation. (**a**) FOXP3^+^ CD4 T cells were measured from no iTreg, media expanded iTreg, and MSC co-culture expanded iTreg treated mice. NSG mice received 10^7^ PBL and iTregs were injected at 7 days after PBL injection. Spleen cells were collected at day 14 after PBL injection. (**b**) FOXP3 expression, as quantified by RT-PCR, in the spleen cells. Measurement of FOXP3^+^ CD4 T cells in iTreg-treated mice. iTregs were expanded in MSC co-culture with CD39 inhibitor vs control. 10 days after expansion, iTreg cells were injected into PBL-treated NSG mice. Spleen cells were collected at day 14 after PBL injection. Expression of IFNγ and IL-17 was measured by flow cytometry in CD4 T cells. (**d**) Measurement of IFNγ producing CD4 and CD8 T cells was performed by flow cytometric analysis. Data are representative of *n* = 3–4 independent samples. *p < 0.05, **p < 0.01, ***p < 0.001 paired t test. See also Supplementary Figs. S8 and S9.

Inhibition of CD39 and CD73 signaling blocked MSC mt transfer to iTregs, and resulted in significantly reduced suppressive function vs. control (Fig. 5f). For assessment of the in vivo suppressive functions of iTreg expanded in MSC co-culture vs. iTreg expanded in standard IL-2/media suspension condition alone, iTregs were adoptively transferred into an NSG xenogeneic GVHD mouse model. iTregs expanded short-term (21 days) were injected 7 days after adult human PBMC were injected to induce GVHD. We observed enhanced survival and reduced lymphocyte infiltration into liver, skin, tongue and intestinal tissues in mice treated with iTregs from MSC co-cultures (Supplementary Fig. 8). RNA levels of FOXP3 and FOXP3^+^ CD4 T cells in harvested spleen were significantly increased in mice treated with MSC co-cultured iTregs at 2 weeks post-GVHD induction (Fig. 7a,b). IFNγ producing CD8^+^ and CD4^+^ T cells were dramatically reduced in spleen cells harvested from mice treated with MSC co-cultured iTregs (Fig. 7d). Consistent with the cytokine staining results, serum and ex vivo levels of pro-inflammatory cytokines IFNγ and TNF were significantly reduced in animals treated with MSC co-cultured iTregs (Supplementary Fig. 9a,b).

To explore the effect of MSC mt transfer on iTreg phenotype and function in vivo, we co-cultured iTregs with MSC in the presence of a CD39 inhibitor^50, 51^. CD39 inhibitor treatment reduced the percentage of FOXP3^+^ iTreg in PBL induced GVHD NSG mice (Fig. 7c). IFNγ producing CD8^+^ and CD4^+^ T cells were significantly increased in mice injected with CD39 inhibitor-treated iTreg (Fig. 7d). IFNγ and TNF were also significantly increased in the serum of these mice (Supplementary Fig. 9c) and ex vivo (Supplementary Fig. 9d). Collectively, these results reveal suppressive iTregs are maintained by MSC mt transfer and can suppress effector T cell immune responses against human inflammatory xeno-immune disease in vivo.

## Discussion

Intrinsic defects in Tregs as observed in autoimmune disease may hamper the success of autologous tTreg adoptive cell therapies^52–54^. Previous studies have shown ex vivo-expanded, partially HLA-matched tTregs from allogeneic UCB sourcing are well tolerated in humans^11^. UCB-derived iTregs exhibit enhanced stability and suppressive function compared to adult blood-derived iTregs in large part due to enhanced expression of the FOXP3-stabilizing transcription factor BACH2^16^. Nevertheless, a major obstacle to the clinical implementation of iTregs is concern over the instability of iTreg FOXP3 expression and loss of suppressor function upon transfer to an inflammatory environment^55^. The results reported in this study demonstrate that MSC co-culture provides enhanced and sustained FOXP3 expression and iTreg suppressive function in inflammatory in vitro and in vivo environments. Importantly, our study provides insight into normal physiologic mechanisms to sustain human proliferating T-cells including mt transfer from adjacent MSC.

Co-culture of iTregs with MSC feeder cells significantly improved the number and suppressive function of iTreg during 21 day IL-2 based ex vivo expansion. MSC co-culture increased iTreg expression of CD25, CTLA-4, and ICOS, while expression of LAG-3 and TIM-3, associated with exhaustion of activated T cells^56–62^, was decreased. We observed higher proportions of CD62L^+^ and absolute numbers of CD45RA^+^ iTreg cells after 21 day IL-2 expansion co-culture on MSC feeder cells. Inflammatory environments exert rapid loss of FOXP3 expression and reduced suppressive activity of iTregs^63^. Our data show significantly enhanced suppressive function, including inhibition of effector cell activation and maintained FOXP3 stability in MSC co-culture expanded iTregs exposed to inflammatory conditions in vitro and in vivo. It will be important in the future to examine whether epigenetic modification of the Treg-specific demethylated region in the FOXP3 gene occurs, in order to gain further insights into the mechanisms of MSC-enhanced iTreg stability^64^.

Although prior studies point to MSC enhancement of Treg function to be primarily via paracrine mechanisms^65–67^, our study shows MSC support of iTreg FOXP3 expression and sustained suppressive function requires cell–cell contact mt transfer. Recent work showed umbilical cord-derived MSC mt can promote adult PBMC CD4 T cell differentiation into iTreg^68^. Intriguingly, purified MSC mt could recapitulate the effects of MSC mt transfer to promote Treg differentiation. However, this study did not examine stabilization of iTreg FOXP3 expression and maintenance of naïve phenotype mediated by MSC mt transfer during ex vivo expansion. Our findings suggest direct cell–cell contact and mt transfer via TNTs play an important role in stabilization of iTreg FOXP3 expression, maintenance of naïve phenotype, and maintained iTreg suppressive function in adverse inflammatory conditions. MSC co-culture was also observed to support maintenance of tTreg phenotype. Notably however, the proportion of FOXP3^+^ tTregs was less sustained compared to iTregs during expansion (57% vs 96%, respectively at Day 21 in MSC co-culture) potentially reflecting the presence of contaminating effector cells and the more stable phenotype of iTregs generally.

Several mechanisms have been proposed to explain how mt may promote Treg formation. For instance, it was proposed retrograde mitochondria to nuclear signaling alters the expression of nuclear genes involved in Treg phenotype via epigenetic mechanisms^68^. However, prior to our studies, the cellular and molecular mechanisms driving MSC mt transfer to proliferating cells and signaling pathways leading to altered phenotype of recipient cells have not been elucidated. Our studies identify iTreg CD39/CD73 signaling drives MSC mt transfer and further mt transfer results in augmented iTreg BACH2 and SENP3 expression. BACH2 regulates human UCB iTreg development via direct transcriptional activity at the FOXP3 promoter, and by suppressing TCR induced gene transcription^16, 69^. SENP3 is a SUMO specific protease known to maintain Treg stability^17^. SENP3 activity can be regulated by a variety of cell stressors including but not limited to ROS^70^. In addition, SENP3 has been linked to mitochondrial signaling mechanisms and function. SENP3 can deSUMOylate and modulate the localization and function of Drp1, a key regulator of mitochondrial fission in neurons. Prior studies have also identified a role for mitochondrial regulation of BACH2 function and cell fate decisions in B cells^49^. Thus, our findings suggest a potential mechanism whereby mitochondrial transfer stabilizes iTreg suppressor function via altered expression of SENP3 and BACH2.

CD39 and CD73 together play strategic roles in immune responses^44, 45^. CD39 and CD73 degrade extracellular ATP to yield AMP and anti-inflammatory adenosine^71^. CD73−/− mice show enhanced antitumor immunity^72^ and more severe gastritis compared to functional CD73 controls, and adoptive injection of WT Tregs reversed these immune responses^42^. Prior studies^73, 74^ identify CD73 signaling on Tregs is critical to maintain Treg suppressive function. Our study identifies MSC mt transfer to iTregs contributes to enhanced iTreg phenotypic stabilization. In addition, the signaling mechanisms involved in mt transfer to Tregs have not been previously examined. We identify CD39 and CD73 signaling mediates MSC mitochondrial transfer to proliferating iTregs during IL-2 driven ex vivo expansion. Further studies on CD39 and CD73 mediated mt transfer may elucidate mechanisms that may be exploited for Treg functional stabilization.

MSCs mt transfer has been shown in several cell types, including proliferating acute leukemia blasts in the marrow microenvironment^39^. Molecular pathways of mitochondrial transfer have been identified, including the mitochondrial Rho-GTPase 1 Miro1^40^. Miro1 regulates intercellular mt transfer by attaching mitochondria to KLF5 the kinesin motor protein^75, 76^ and has been implicated in enhanced injured cell recovery^40^. In one study, injured astrocytes induced increased levels of Miro1 expression in MSC and this was correlated with mt transfer^20^. However, MSC mt transfer to Treg cells and the cellular and molecular mechanisms underlying mt transfer has not been previously identified. Our data show mitochondrial metabolic function through CD39/CD73 signaling, in proliferating iTregs induced Miro1 expression in MSC during co-culture ex vivo. MSC mt transfer occurs via TNT rather than exosome mediated transfer. These findings provide impetus to study the cell signaling mechanisms in proliferating iTreg serving to induce MSC Miro1 expression and facilitate TNT formation for mt transfer.

Mitochondria are an important source of ROS^77^ and regulate cell cycle, modulate signal transduction^78, 79^, and are critical for cancer cell tumorigenicity^80^. This is particularly relevant considering ROS is a major driving force for mt transfer via TNTs from bone marrow stromal cells to leukemic blasts^39^. Further work demonstrated mt metabolism plays a critical role in T cell activation. Thus, future studies are warranted to investigate whether the transfer of MSC mt modulates these metabolic signaling pathways in proliferating Treg. Our observations strongly indicate increased mt quantity in iTregs was derived from MSCs via TNT transfer. However, ROS was not a critical mechanism driving this process. Nonetheless, cytochalasin B treatment led to diminished number of FOXP3^+^ iTregs, suggesting TNT transfer of mt from MSC play an important role in maintenance of FOXP3^+^ iTreg stability.

Regardless of cell type, mt are now recognized to play roles extending well beyond the production of energy. T cells undergoing changes in cellular metabolism during activation is a central idea emerging from many recent studies^81^. In contrast to quiescent T cells, with limited metabolic needs mainly encompassing cellular trafficking and housekeeping functions, actively proliferating cells must generate additional ATP for functions including the generation of intermediates required in various biosynthetic pathways and signaling molecules for anabolic metabolism. Future work is needed to identify how signaling mechanisms can be modulated to modulate mt transfer. Importantly, several lines of evidence support mt transfer is a key mechanism of MSC supportive function. Our studies identify iTreg mt membrane potential is enhanced by culture over MSC feeder cells, and further pharmacologic inhibition of mt transfer nearly completely negates the benefit of MSCs on iTreg FOXP3 expression and suppressive function.

In summary, our findings highlight the importance of mt function in maintaining iTreg stability in MSC co-culture expanded iTreg and provide insight into cellular and molecular mechanisms driving MSC mt transfer to proliferating iTreg. This process inhibits differentiation, cell senescence, maintains robust FOXP3 expression, as well as suppressive function despite an adverse inflammatory milieu in vitro and in vivo. Although several factors modulate iTreg stability and function in disease models, our results show the mt BACH2 pathway is a prominent mechanism of MSC-enhanced iTreg stability and function. Additionally our work highlights the important role played by CD73-regulated TNT formation that serves to facilitate mt transfer into proliferating iTreg during expansion.

## Methods

### Mice

NSG mice were ordered from Case Western Reserve University (CWRU) a thymic facility. Mice were maintained in the SPF animal facility at CWRU. Mice were ear-punched, weighed, placed in 12-pie irradiator holder and exposed to 240 rads from Gammacel^137^ Cs source.

### Human bone marrow MSC culture

De-identified bone marrow samples were obtained from human BM aspirate from the CWRU Biorepository. Briefly, marrow mononuclear cells were resuspended at a concentration of 2 × 10^6^ cells/ml in completed IMDM (GIBCO BRL Life Technologies, Grand Island, NY, USA) medium. Media contained 20% heat-inactivated human serum albumin (Gemini Bio-product, Sacramento, CA) with 1% penicillin and streptomycin (Gibco), 40 ng/ml basic fibroblastic growth factor (Sigma). Cells were cultured in 175 cm^2^ flasks and incubated at 37 °C 5% CO_2_ in a humidified incubator for 2–4 weeks until a confluent layer was formed. BM-MSCs were characterized by analyzing the expression of CD markers (CD11b, CD19, CD34, CD44, CD45, CD73, CD90, CD105, and HLA-DR; BD Stemflow hMSC Analysis Kit; BD Biosciences, San Jose, CA, USA) by flow cytometry. MSCs between passages 3–5 were used in experiments.

### Human UCB and adult peripheral blood samples

Acquisition of de-identified volunteer young adult peripheral blood and UCB were obtained with written informed consent per Case Western Reserve University/Cleveland Cord Blood Center IRB-approved protocols. All methods in these studies were carried out in accordance with relevant guidelines and regulations. Mononuclear cells were isolated by Ficoll-Paque PLUS (GE Healthcare Life Sciences, Piscataway, NJ) density gradient centrifugation with SepMate-50 tubes (STEMCELL Technologies, Vancouver, BC, Canada).

### FOXP3^+^ iTreg generation and ex vivo expansion

UCB derived FOXP3^+^ iTregs cells were generated as previous described^82^. Ex vivo expansion of FOXP3^+^ iTregs was performed with CD25^+^ cells MACS purified from day 4 TGFβ-induced UCB iTreg. On Day 4 of TGF-β conditioning, CD25 + iTregs were MACS purified, rested for 48 h, and on Day 6, 5 × 10^5^ purified cells were split into expansion cultures with either media containing 100 U/ml IL-2 in suspension or identical media/IL-2 over MSC feeder cells. Cultures were fed every other day with fresh media/IL-2 and MSC during short-term culture (21–28 days). No re-stimulation was performed during the 21–28 day expansion following the initial 4 day stimulation consistent with a previously described UCB Treg expansion protocol^83^. For isolation of UCB-derived tTregs, EasySep Human CD4 + CD127lowCD25 + Regulatory T Cell Isolation Kit (Stem Cell Technologies) was used. tTregs were maintained under identical conditions as iTregs. Absolute number of FOXP3^+^ iTreg cells were calculated from the percentage of CD4^+^ cells based on the total viable cell count obtained by trypan blue dye exclusion at each indicated time point. For iTreg stability assays, harvested iTregs were stimulated with CD3 and CD28 antibodies (1 μg ml^−1^) with IFNγ (10 μg ml^−1^), IL-6 (10 μg ml^−1^), and TNF (10 μg ml^−1^) at concentration of 5 × 10^5^ cells ml^−1^ for 72 h and analyzed.

### Flow cytometry analysis

Cells were harvested on days 7, 14, and 21 of iTreg expansion, and were surface stained with antibodies against CD4-FITC, PECy7 (RPA-T4), CD8-FITC (RPA-T8), CD25-PE (M-A251), CD45RA-PE CF594 (HI100), CD45RO-PE (UCHL1), CD62L-BB515 (SK11), CD223 (LAG-3)-BV421 (T47-530), CD304 (NRP-1)-Alexa Fluor 647 (U21-1283), CD278 (ICOS)-PECy7 (398.4A), CD279 (PD-1)-PE (EH12.1), CD366 (Tim-3)-Alexa Fluor 647 (7D3) (BD Bioscience, San Diego, CA). For CTLA-4 and FOXP3 cytoplasmic staining, APC anti-CTLA-4 (BNI3, BD pharmingen) and PE anti-FOXP3 (3G3, Miltenyi, Auburn, CA, USA) antibodies were utilized. For intracellular staining, cells were fixed after surface staining and permeabilized with Fixation/Permeabilization kit according to the manufacturer’s protocol (Miltenyi). A Fortessa instrument (BD Biosciences) was used for data acquisition and the data was analyzed using FlowJo software (Tree Star, Inc., Ashland, OR). To measure intracellular cytokines, cells were stimulated for 4 h with PMA (10 ng/ml; Sigma-Aldrich) and Ionomycin (1 μM) in the presence of 2 μM Monensin (Calbiochem; San Diego, CA) during the last 2 h of incubation and fixed with 4% paraformaldehyde, permeabilized, and stained with fluorochrome conjugated antibodies.

### Suppression assay

In vitro suppression assays were performed as previously described^82^. At 3 weeks of expansion, CD25^int^ FACS sorted iTregs were used for suppression assays. Responder naïve CD4^+^ T-cells and Mitomycin C treated T-cell depleted PBMC were prepared from a healthy donor. CFSE labeled naïve CD4^+^ T-cells were plated at a 1:1 ratio with Mitomycin C treated T-depleted PBMC and varying concentrations of UCB iTreg. Soluble anti-CD3 (2 μg/ml) (clone HIT3a, BD Bioscience) mAb was added. Suppression was measured by cell division index by FACS.

### Measurement of mt transfer

For mt and cell labeling, MitoTracker Green, MitoTracker Red FM, and CellTrace Far Red were purchased from Thermo Fisher Scientific. MSCs were incubated in MitoTracker Green (200 nM) or MitoTracker Red FM (500 nM) for 45 min and washed 3 times with pre-warmed media and iTreg cells were incubated in CellTrace Far Red and stained according to the manufacturer’s instructions. MSC and iTreg were then cocultured for 24–36 h and then assessed via flow cytometry or imaged at indicated time periods on 35 mm #1.5 glass bottomed petri dish (Cellvis; Mountain View, CA) using a Zeiss Axiovert microscope equipped with Apotome.2 using the 63 × oil immersion objective. Analysis of recorded images was performed using Zen 2012 software.

### Quantitative real-time polymerase chain reaction

Total RNA was extracted from cells using GeneJet RNA Purification Kit (Thermo Scientific, Waltham, MA) and quantified. cDNA was subsequently synthesized using High Capacity cDNA Reverse Transcription Kits (ThermoFisher). Real time quantitative PCR was performed using gene-specific primers and probe sets (Applied Biosystem, Foster City, CA) and a QuantStudio 3 RT-PCR instrument (Applied Biosystems). The qRT-PCR runs were performed in triplicate to quantify expression levels for each gene using Taq-man assays per manufacturer’s instructions.

### ROS measurement

To measure mt ROS production, 1 × 10^6^ cells were incubated in complete media with 5 μM MitoSox (Invtirogen, Carlsbad, CA) for 30 min at 37 °C. Cells were washed with pre-warmed media and FACS buffer. Cells were stained with relevant surface Abs for FACS analysis. To measure total ROS, 1 μM 2′,7′-dichlorofluorescin diacetate (DCFDA) was used. Data were acquired on a Fortessa (BD Bioscience) and analyzed using FlowJo (Tree Star).

### Mitochondria mass quantification

To quantify mt DNA copy number, we used Terra qPCR direct polymerase mix and Human Mitochondria DNA (mtDNA) monitoring primer set (Takara Bio USA) according to the manufacturer’s instructions.

### Western blot analysis

Collected cells were lysed using radio-immune precipitation assay (RIPA) buffer and protein was quantified using the Bradford assay (BioRad, Hercules, CA), with 40 μg of protein separated by 4–20% SDS-PAGE (BioRad). Proteins were electro-transferred onto Immobilon membrane (Millipore) and were probed with polyclonal rabbit anti-BACH2 (D3T3G; Cell signaling technology, Beverly, MA) anti-SENP3 (D20A10; Cell signaling technology) antibody, monoclonal rabbit anti-FOXP3 (D25D4; Cell signaling technology), and monoclonal mouse/human/rat anti-β-actin antibody (R&D systems, Minneapolis, MN). Applicable HRP-conjugated secondary antibodies were used with ECL chemi-luminescence to observe relevant bands using ChemiDoc XRS + image system (BioRad). The band intensity was quantified using Image J.

### Cytoplasmic and nuclear protein isolation

Cytoplasmic and nuclear fractions were isolated from ~ 10–20 × 10^6^ Treg cells from IL-2/media only cultures or Tregs from IL-2/media MSC co-cultures using the NE-PER Nuclear and Cytoplasmic extraction reagents kit (Invitrogen 78833) according to the manufacturer's instructions. In brief, Treg cells were removed from co-culture, washed with PBS, and ice cold CER1 reagent was added, and samples were pelleted by centrifugation. Samples were vortexed for 15 s and incubated on ice for 10 min. Ice cold CERII reagent was added and samples were vortexed for an additional 5 s and incubated on ice for 1 min. After additional 5 s vortex, samples were centrifuged for 5 min at maximum speed. The supernatant containing the cytoplasmic extract was transferred to a new tube. The insoluble pellet fraction containing the nuclei was suspended in ice-cold NER and vortexed for 15 s every 10 min for 40 min. Nuclear fraction was centrifuged at maximum speed for 10 min and supernatant containing the nuclear extract was transferred to a new tube. Isolated fractions were kept on ice. Protein concentration was determined by Bradford assay and equivalent amount of total protein for each sample (5–10 μg nuclear fraction, 20–40 μg cytoplasmic fraction) was loaded onto a 4–20% SDS-PAGE gel and processed for analysis of BACH2 and SENP3 expression by western blotting as described above.

### 5-Bromo-2′-deoxyuridine (BrdU) assay

10 μM BrdU was added to media or MSC co-culture iTreg expansion culture well during the last 48 h. After 2 h incorporation, iTreg cells were harvested and BrdU staining was performed per manufacturer’s instructions (Invitrogen). Cells were stained with CD4 to exclude non iTregs.

### pLV-mitoGFP Lentivirus production and transduction

Lentiviral plasmids pLV-eGFP and pLV-mitoGFP were gifts from Pantelis Tsoulfas (Addgene plasmids # 36083 and 44385)^84, 85^. Lentiviral plasmids were isolated from Stbl3 cells by minipreps (Qiagen) of 5 mL overnight cultures established from single colonies. Lentivirus was produced in HEK293T cells. Transduction experiments were performed by spinoculation transduction method as described^86^.

### ELISA assay

Levels of IFNγ and TNF were detected from serum and culture supernatant. IFNγ and TNF were determined by ELISA (R&D systems), according to the manufacturer’s instructions.

### GVHD model and tissue histology

For establishment of an acute GVHD model, NSG mice received cord blood CD34^+^ cells. 5–6 weeks later, mice were injected with adult PBL (1 × 10e7). GVHD severity was assessed by an established scoring system based on weight loss, posture, activity, fur texture and skin texture^87^. Pathologic evaluation was performed for organs targeted by acute GVHD, including liver, skin, tongue and intestine^88^. Tissues were collected and hematoxylin/eosin (H&E) stained at day 21 after injection of PBL. Intestines were washed thoroughly prior to sample collection. All samples were fixed using 10% neutral-buffered formalin for 24 h and embedded in paraffin. Paraffin sections (1 μm) were cut, dewaxed and stained with H&E^89^.

### Statistical analysis

Statistical comparative analyses were performed using the Paired *t*-test (Prism 7 software-GraphPad, San Diego, CA). Multiple comparisons analysis was performed using the Friedman test. Data are presented as the mean ± standard deviation, SD. A p value of < 0.05 was considered significant. *p < 0.05, **p < 0.01, ***p < 0.001.

### Study approval

This study was designed to determine whether MSCs can enhance the stability and suppressive capacity of iTregs during ex vivo expansion. MSC were obtained from healthy donor derived bone marrow and umbilical cord blood from at least 3 different donors to confirm our observation of MSC mitochondrial transfer mediated stabilization of iTregs. The study was carried out in accordance with the approved guidelines and regulations. De-identified human cells tested in this study were approved by Cleveland Clinic Foundation for volunteer umbilical cord blood collection with written informed consent from donating mothers. In addition, the collection of normal adult peripheral blood from de-identified human donors was approved by the Case Western Reserve University IRB for the Case Comprehensive Cancer Center Hematopoietic Stem Cell Facility (CASE 12Z05). For GVHD studies, iTregs expanded in either media/IL-2 suspension culture condition or media/IL-2 over MSC feeder cells, were harvested and injections were performed by a second investigator (DA) who was blinded to the identity of each sample. All animal experiments and methods were performed in accordance with the relevant guidelines and regulations approved by the Institutional Animal Care and Use Committee of Case Western Reserve University. In addition, mice studies were carried out in compliance with the ARRIVE guidelines. Mice were randomized on the basis of their age, sex and weight. Numbers of mice were chosen based on magnitude of observed effects and variability in the results and are indicated in figure legends.

## Supplementary Information

Supplementary Information.

## Data Availability

All data generated or analyzed during this study are included in this published article and its Supplementary Information files.
