# Supplementary material for: Mesenchymal stromal cell mitochondrial transfer to human induced T-regulatory cells mediates FOXP3 stability
Source: Sci Rep. 2021 May 21;11:10676. doi: 10.1038/s41598-021-90115-8 (PMC8140113; doi:10.1038/s41598-021-90115-8)

Supplementary Information for

Mesenchymal stromal cell mitochondrial transfer to human induced T-regulatory cells mediates FOXP3 stability

Jeong-su Do[1*], Daniel Zwick[2], Jonathan D. Kenyon[2], Fei Zhong[2], David Askew[3], Alex Y. Huang[3], Wouter Van't Hof[2], Marcie Finney[2], and Mary J. Laughlin[2,4]

[*] To whom correspondence should be addressed: Jeong-su Do, PhD, Department of Immunology and Theranostics, 1500 E. Duarte Road, Beckman Research Institute, Duarte, CA 91010. TEL) 626-218-2748; EMAIL) jedo@coh.org

[1]Diabetes and Metabolism Research Institute, The Beckman Research Institute, City of Hope National Medical Center, 1500 E. Duarte Road Duarte, CA 91010 USA.
[2]Cleveland Cord Blood Center, 25001 Emery Rd, Cleveland, OH 44106 USA.
[3]School of Medicine, Department of Pediatrics, Case Western Reserve University, Cleveland, OH 44106 USA.
[4]School of Medicine, Department of Biomedical Engineering, Case Western Reserve University, Health Education Campus, 9501 Euclid Ave, Cleveland, OH 44106 USA.

Figure S1

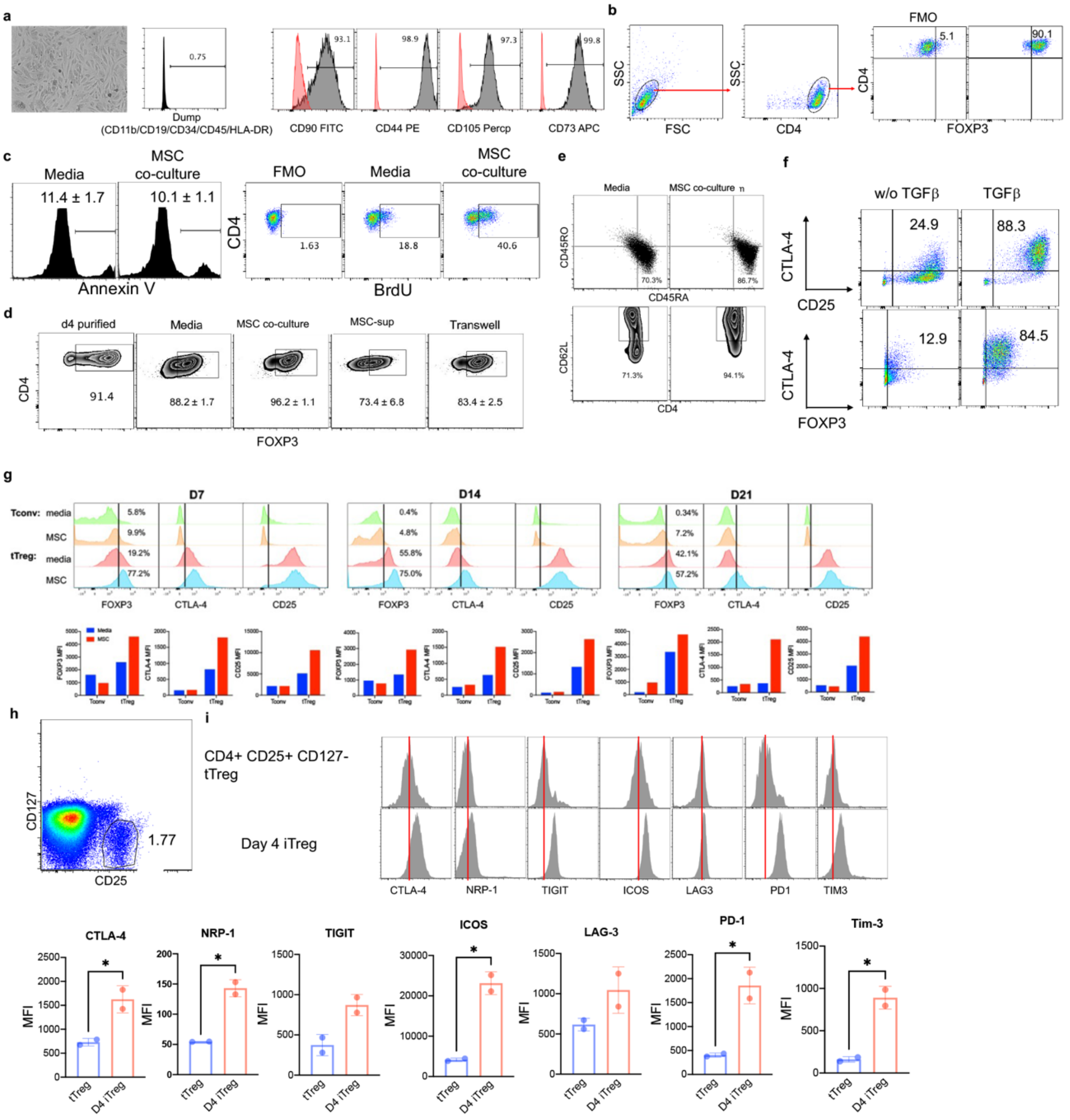

Supplemental Figure 1. MSC characterization and iTreg phenotype and stability

(**a**) Representative morphology and flow cytometric analysis of MSC phenotype. MSC were trypsinized at passage number 3 and FACS analysis was carried out for MSC surface markers CD90, CD44, CD105, and CD73 with isotype control

(red). **(b)** Gating strategy for FOXP3+ iTregs. On Day 4, cells were collected and stained with CD4 APC and FOXP3 PE. FOXP3 expression was analyzed vs. fluorescence minus one (FMO) control (**c**) Naïve CD4 T cells were differentiated to FOXP3+ iTreg cells and expanded in IL-2/media with or without MSC feeder cells. Cells were stained for Annexin V and FACS analysis was performed at day 21. At day 21, iTregs were BrdU pulsed (10 μM) for the last 1 h of culture. BrdU incorporation was analyzed by FACS (**d**) Frequency of $FOXP3^+$ iTregs**.** Data represent FOXP3 expression on UCB iTregs at day 21 of expansion. (**e**) Representative flow cytometric plots of CD45RA and CD62L expression on iTregs expanded in IL-2/media or identical IL-2/media over MSC monolayer. Flow cytometry data are representative of 3 independent experiments (*n* = 6-7). **(f)** FACS analysis of iTregs after 4 days differentiation. Naïve CD4 T cells were stimulated w/ or w/o TGFβ. 4 days later, T cells were harvested and stained with CD4/CD25/FOXP3/CTLA-4**.** (**g**) MACS purified conventional and tTreg cells were expanded in IL-2/media alone or identical IL2/media over a MSC feeder layer. Cells were collected at indicated time points for FACS analysis. (**h**) Cord Blood tTreg cells were gated as CD4+CD25+ CD127-. (**i**) Cord blood naïve CD4 T cells were stimulated in iTreg induction condition. 4 days after stimulation cells were harvested and stained with indicated antibodies. MFI analysis of tTreg vs D4 differentiated iTreg cells.

Figure S2

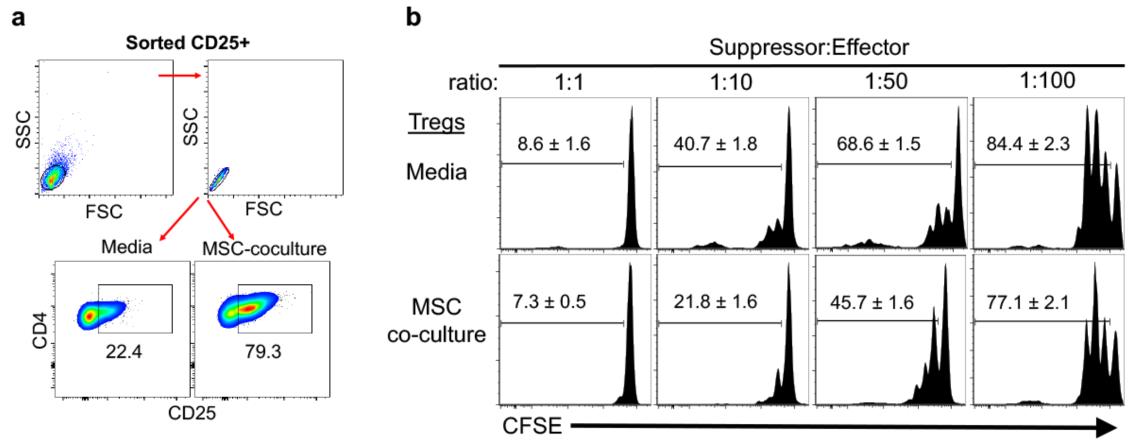

Supplemental Figure 2. Suppressive effect of iTregs

(**a**) Gating strategy for sort of $CD25^+$ iTreg cells for suppression assay. (**b**) Flow cytometric analysis for CFSE dilution-based suppression assay. At 3 weeks post ex vivo expansion of iTregs in IL-2/media vs IL-2/media/MSC co-culture, $CD25^{high}$ Tregs were FACS sorted and assessed for suppressive function. Data are representative of 3 independent experiments.

Figure S3

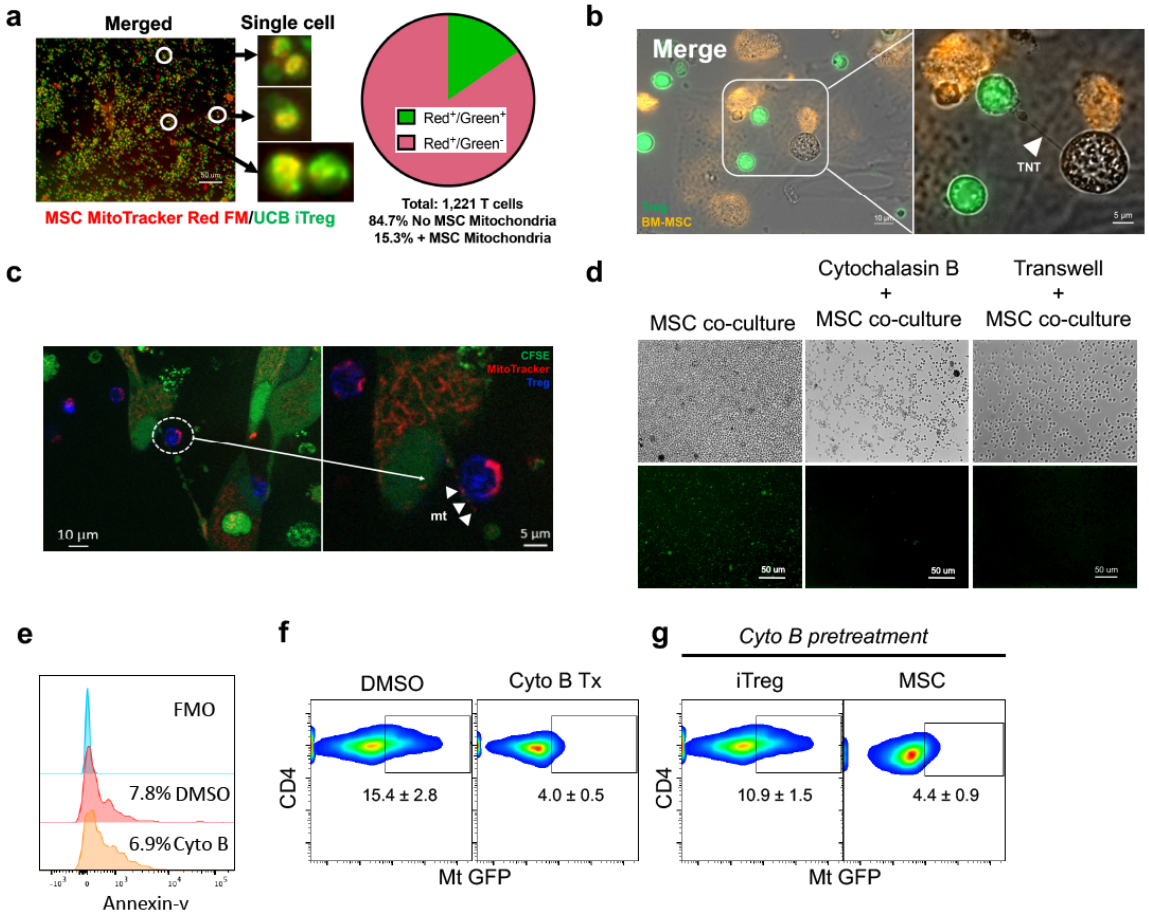

Supplemental Figure 3. Confocal microscopic analysis of MSC mitochondrial transfer to iTregs via TNT

(**a**) Measurement of MSC transfer of mitochondria (labeled with MitoTracker Red) to iTregs (CFSE; green). Cells were co-incubated and images were taken with an Axio Observer-Z1 microscope. Recorded images were obtained using Zen 2012 software. Data show one representative of 4 independent experiments. (**b**) Analysis of iTreg-MSC interactions via TNTs. iTregs were stained with CFSE (green) and MSC were pre-stained with PKH26 (orange) and then co-cultured for 24h. Images were taken with an Axio Observer-Z1 microscope. (**c**) Analysis of

MSC mitochondrial transfer to iTreg via TNT. MSC were pre-stained with CFSE and MitoTracker Red FM and then cultured with Hoechst stained iTreg for 24 h. After 24 h, images were obtained. Analysis of recorded images was performed using Zen 2012 software. Images are representative of 4 independent experiments ($n = 6$). (**d**) Measurements of MSC mitochondrial transfer in the presence of TNT inhibitor and indirect transwell co-cultures. After 24-36 h incubation, iTregs were harvested by gentle wash, MSC were stained with MitoTracker Green FM and images were collected on an Axio Observer-Z1 microscope. (**e-g**) Effect of iTreg vs. MSC pre-treatment vs. co-treatment with Cyto B on mt transfer and iTreg viability. TGF-β derived UCB FOXP3+ CD4 T cells were treated with DMSO or Cyto B. (e) 72 h after iTreg treatment with Cyto B, iTregs were stained with Annexin V. (f) Effect of DMSO or Cyto B added to iTreg/MSC co-culture on mt transfer. (g) Effect of iTreg or MSC (mito-GFP) pretreatment with Cyto B (prior to co-culture) on mt transfer.

Figure S4

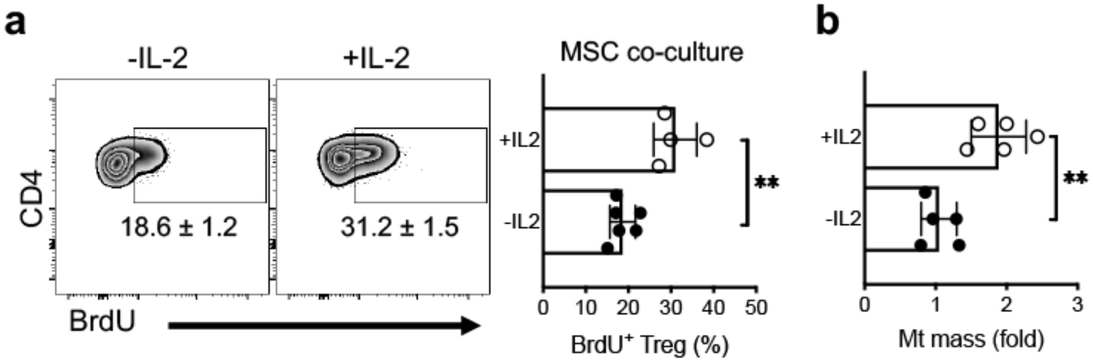

Supplemental Figure 4. Enhanced mt mass in proliferating iTregs

(**a**) Representative flow cytometric plot of BrdU incorporation assay for iTreg proliferation with/without IL-2. iTreg were pulsed with BrdU (10 μM) during the final 2 h of culture. BrdU incorporation was analyzed by FACS. Data are representative of two different experiments and represent the mean ± SD (*n* = 4-6). (**b**) Mitochondrial DNA copy number was assessed from MSC co-culture expanded iTregs with (proliferating)/without IL-2 (quiescent) (n = 5-6). Mitochondrial DNA was collected from iTregs at 21 days post expansion. ** p < 0.01 unpaired Student's t test.

Figure S5

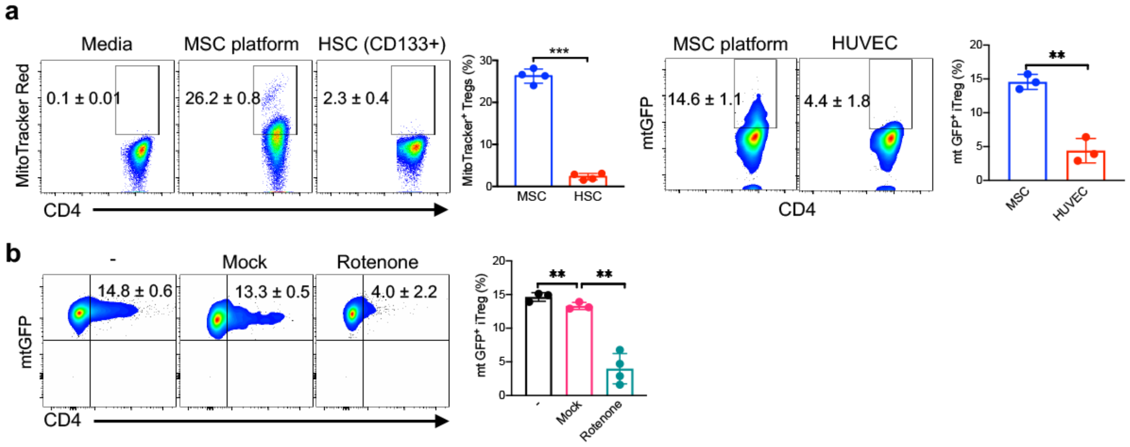

Supplemental Figure 5. Mitochondrial transfer by MSC into proliferating iTregs

(**a**) Representative flow cytometric plot of mt transfer into proliferating iTregs. iTreg were co incubated with MitoTracker stained MSC or HSC. Mitochondria transfer was analyzed by FACS. Data are representative of two different experiments and represent the mean ± SD (n = 3-4). (**b**) Flow cytometric analyis of the effects of rotenone treatment on MSC mtGFP transfer to iTregs. mtGFP^{+} MSCs were pretreated with rotenone (200 nM) at 37 °C for 20 min. mtGFP+ MSCs were then co-cultured with iTregs and mtGFP^{+} iTregs were analyzed after 24 hrs. Data are representative of two different experiments (n=3-4). **$p < 0.01$, *** $p < 0.001$ unpaired Student's t test.

Figure S6

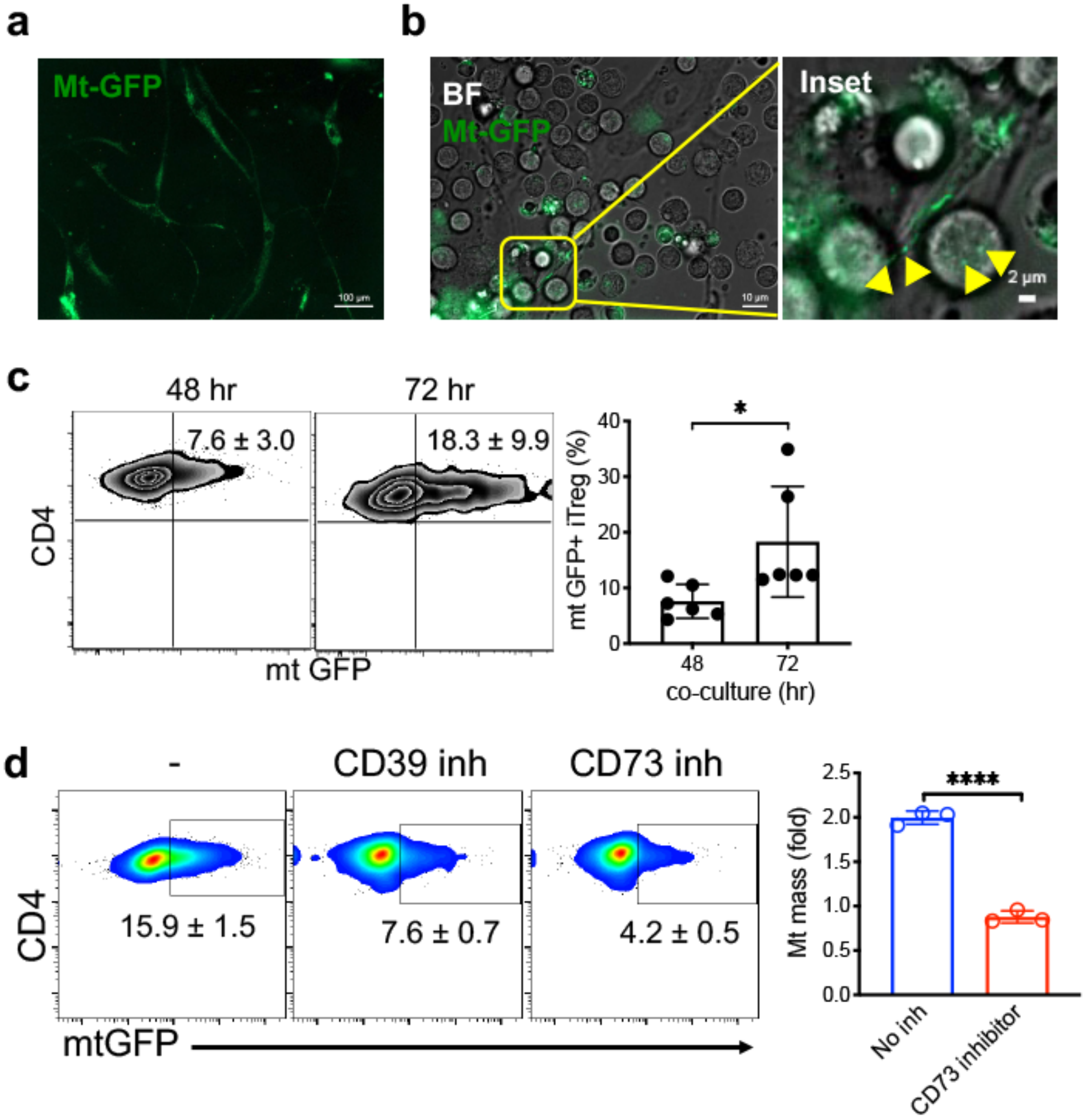

Supplemental Figure 6. CD39/CD73 signaling drives MSC mitochondrial transfer to proliferating iTregs

(**a**) Image analysis of MSCs transduced with mtGFP lentivirus. Images were obtained 72h post-transduction using a Zeiss Axiovert Z1 microscope. (**b**) Image analysis of MSC transfer of GFP$^+$ mitochondria to proliferating iTregs. mtGFP$^+$ MSCs were co-cultured with iTregs and images were collected on a Zeiss

Axiovert Z1 microscope with Apotome.2 after 72 h. Mt-GFP staining in iTregs was analyzed (inset). (**c**) Flow cytometric plots of mtGFP$^+$ iTregs measured at 48h and 72h of co-culture by FACS. Data are representative of 2 different experiments (*n* = 6). (**d**) Flow cytometric plots of the effects of CD39 and CD73 inhibitor treatment on MSC mtGFP transfer to iTregs. mtGFP$^+$ MSCs were co-cultured with iTregs and mtGFP$^+$ iTregs were analyzed at indicated time points. Mitochondrial quantity was measured from MSC co-culture and MSC/ CD73 (100 nM) inhibitor treated MSC expanded iTregs by RT-PCR (n=3). Data are representative of two different experiments. $^*p < 0.05$; **** $p < 0.0001$ unpaired Student's t test.

Figure S7

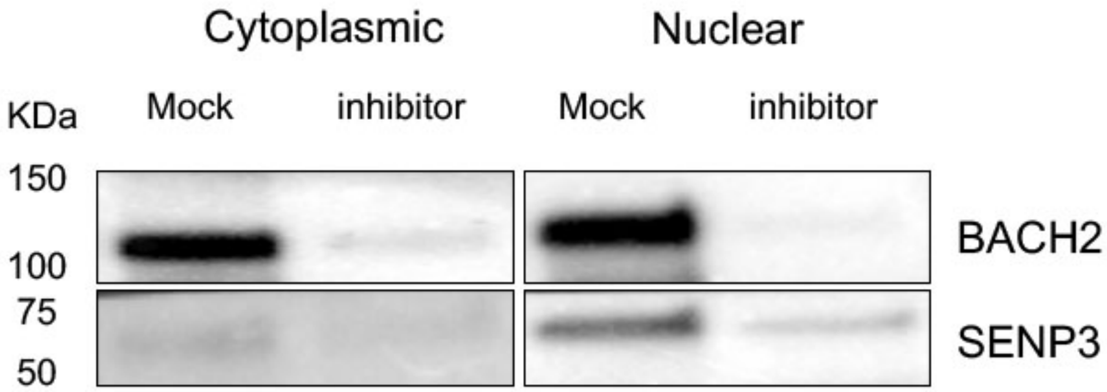

Supplemental Figure 7. ROS signaling promotes BACH2 and SENP3 expression in iTregs during MSC co-culture

Immunoblot analysis of BACH2 and SENP3 expression in lysates of iTregs cultured with MSC in the presence or absence of ROS inhibitor (antioxidant N-acetylcysteine (NAC), 200 μM). Cytoplasmic and nuclear proteins were isolated according to the manufacturer's protocol (Invitrogen). Protein bands were visualized using the ChemiDoc™ XRS+ image system (Bio-Rad).

Figure S8

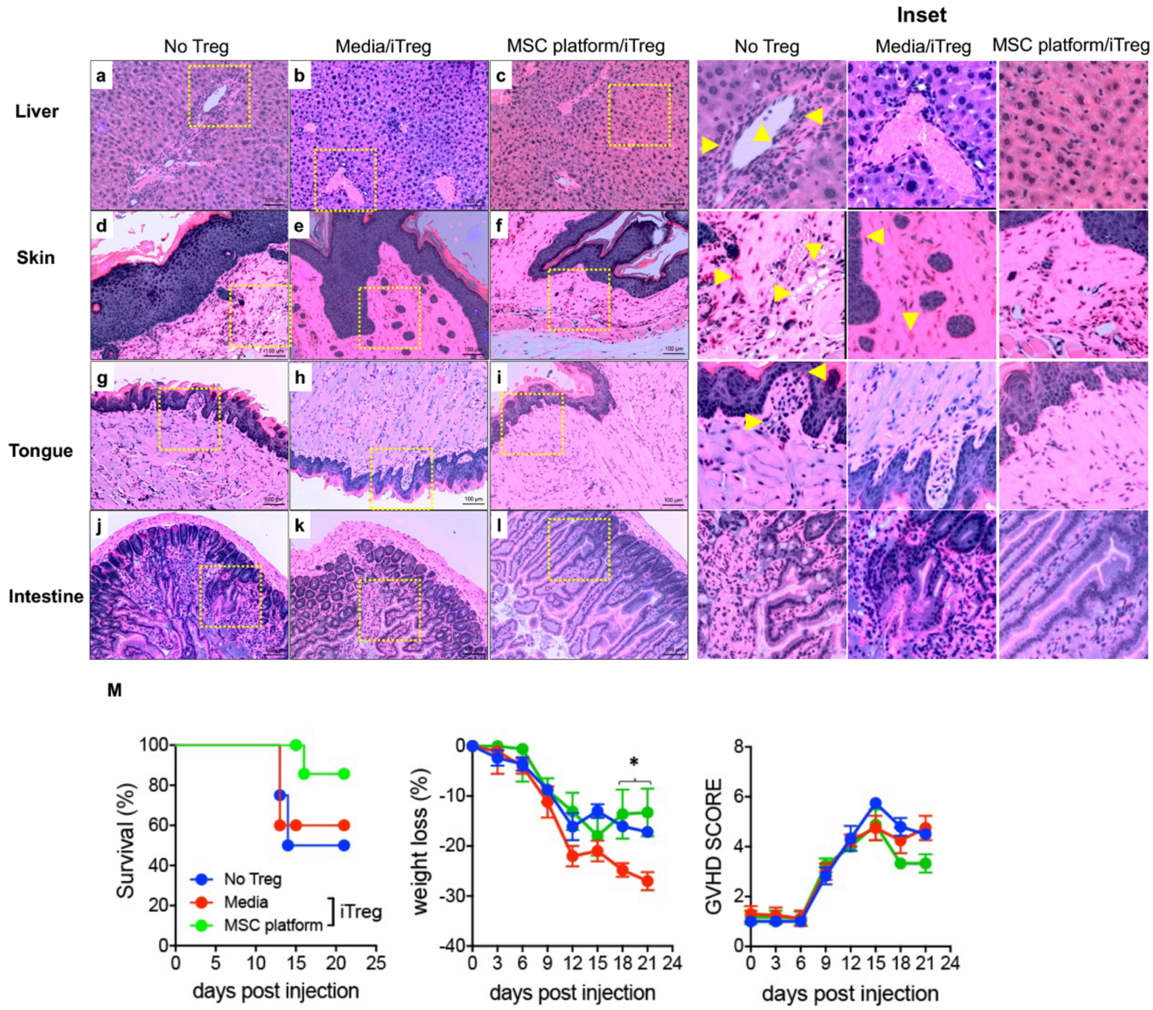

Supplemental Figure 8. Effect of iTregs in GVHD model in humanized mice. NSG mice received cord blood CD34+ cells. 5-6 wks later, mice were injected with adult PBL (1x10e7). (**a-l**) Liver, Skin, Tongue, and Intestine were collected and H&E stained at day 21 after injection of PBL. (**a-c** and **inset**) H&E stained liver sections showing dense lymphocytic infiltrate (arrows) and endothelial inflammation in GVHD mice with no Treg treatment (**a**) which is reduced in mice treated with IL-2/media-expanded iTreg (**b**) and further reduced in mice treated

with iTreg expanded in IL-2/media over MSC (**c**). (**d-f**) H&E stained skin sections showing apoptosis and basal vacuolar damage in keratinocytes (arrows) in GVHD mice with no Treg treatment (**d**) which is reduced in mice treated with IL-2/media-expanded iTreg (**e**) and further reduced in mice treated with iTreg expanded in IL-2/media over MSC (**f**). (**g-i and inset**) H&E stained tongue sections showing epithelial cell apoptosis (arrows) in GVHD mice with no Treg treatment (**g**) which is reduced in Treg/Media injected mice (**h**) and further reduced in Treg/MSC injected mice (**i**). (**j-l and inset**) H&E stained intestinal sections showing apoptosis in the mucosa (arrows) in GVHD mice with no Treg treatment (**j**) which is reduced in Treg/Media injected mice (**k**) and further reduced in Treg/MSC injected mice (**l**). (**m**) Representative survival, weight loss, and GVHD score.

Figure S9

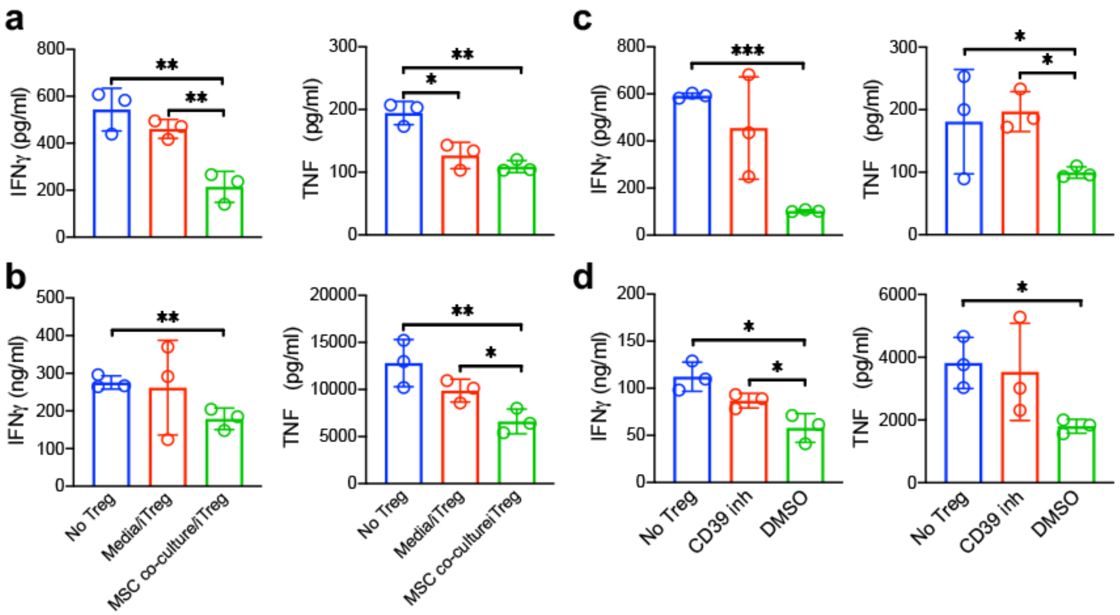

Supplemental Figure 9. Immunosuppressive function of iTregs expanded in MSC co-cultures

Quantification of levels of IFNγ and TNFα from mice that received iTregs expanded in MSC co-culture (**a, b**) or iTregs were expanded in MSC co-culture with CD39 inhibitor vs control (**c**,**d**). (**a**,**b**) Quantification of levels of IFNγ and TNF in serum (**a**) and supernatants (**b**) following stimulation of splenic T cells by PMA (10 μg ml^{-1}) and ionomycin (10 μM). Supernatants were collected at 48h after stimulation. Levels of IFNγ and TNF in serum (**c**) and production from splenic T cells (**d**) were quantified by ELISA. Data are representative of 3 individual mice. *$p < 0.05$; ** $p < 0.01$; ***$p < 0.001$ unpaired Student's t-test.

Figure S10. The images of the original blots for each figure are shown below

Fig. 5d

repeated data of Miro-1 for statical analysis

In the Figure 5d images showing Miro1 Western blots, lanes 1-4 correspond to lanes 4-1 in the original blot image, respectively.

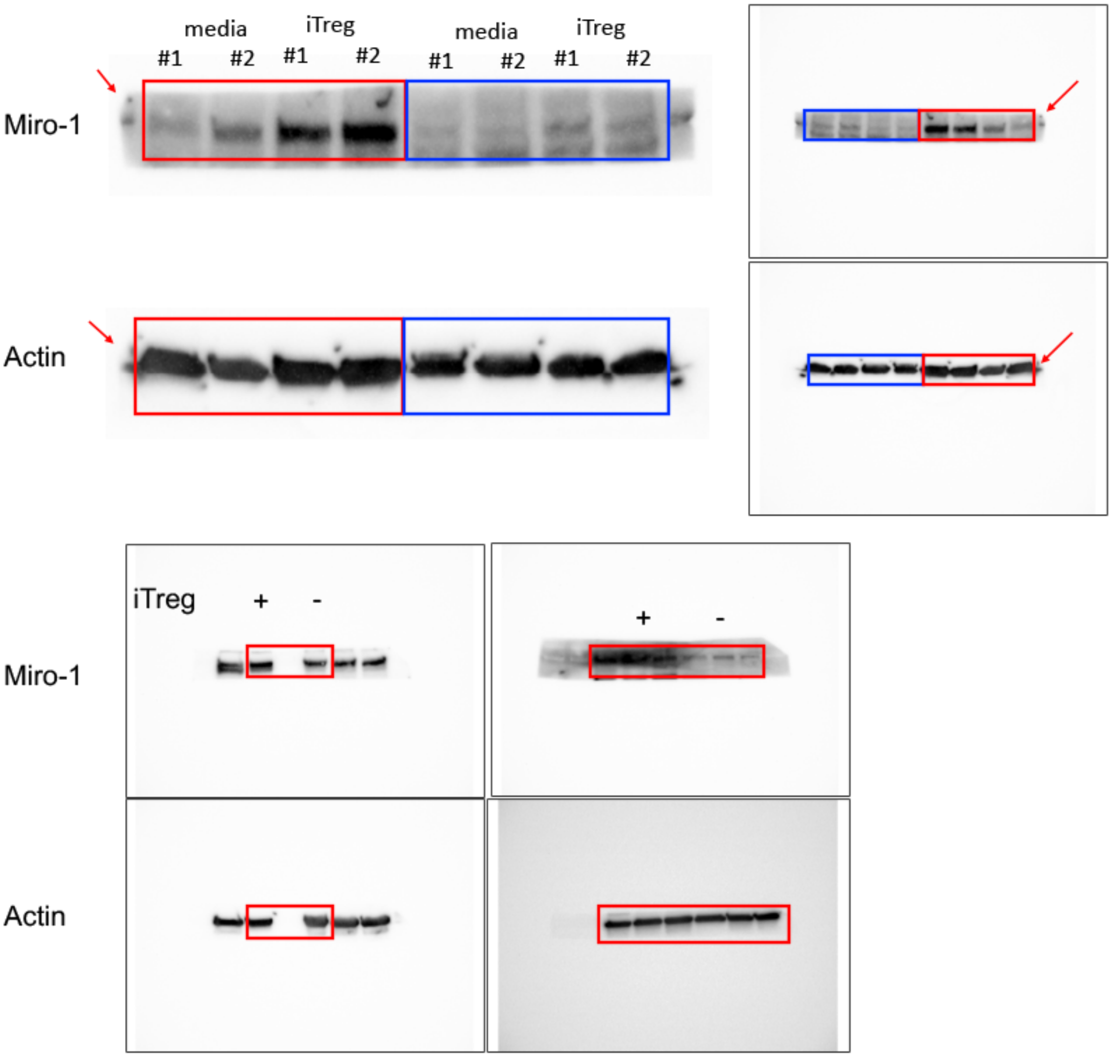

Fig. 5e

repeated data of Miro-1 for statical analysis

In the Figure 5e images showing Miro1 Western blots, lanes 1-4 correspond to lanes 4-1 in the original blot image, respectively.

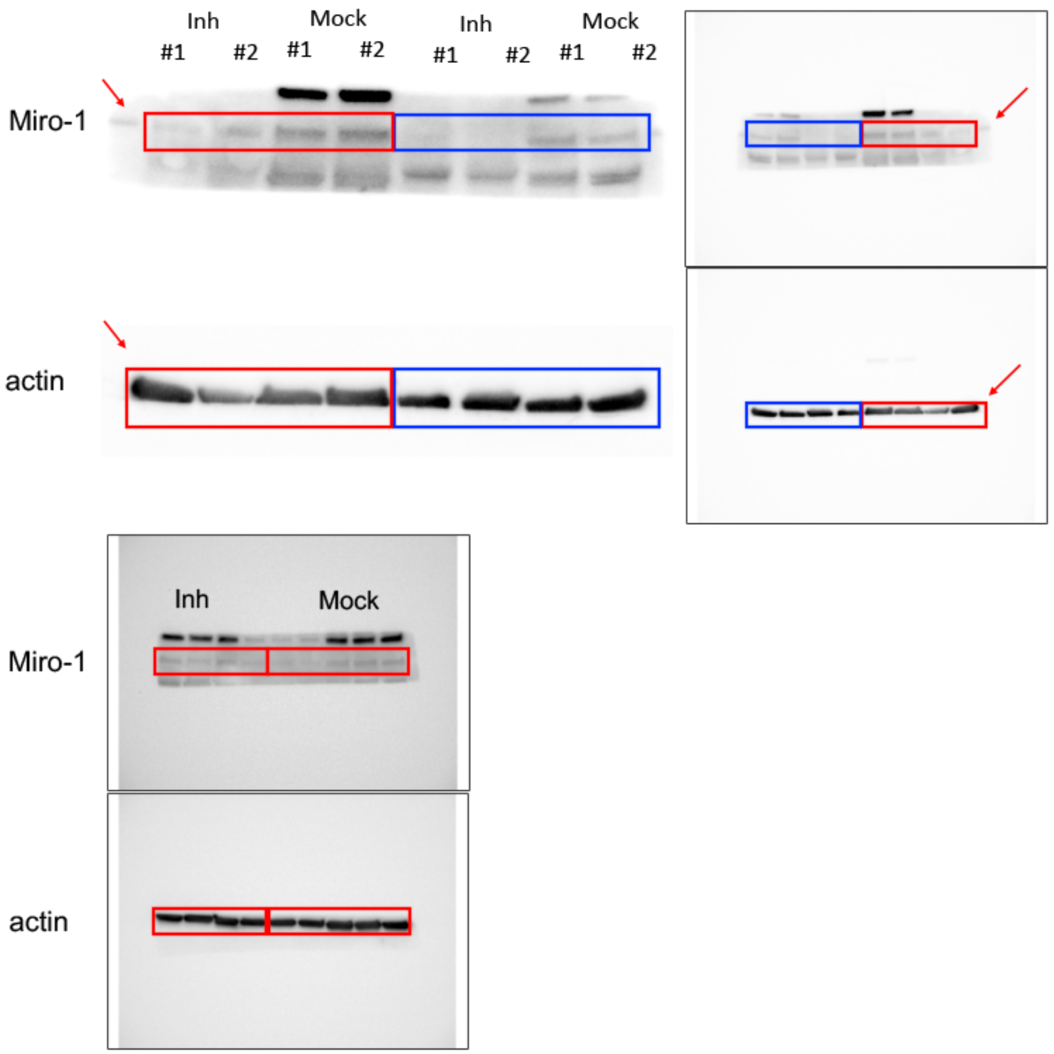

Fig. 6a

repeated data for statical analysis

In Figure 6a image showing BACH2 and FOXP3 western blot, lanes 1-4 correspond to lanes 3-6 in the original blot image, respectively

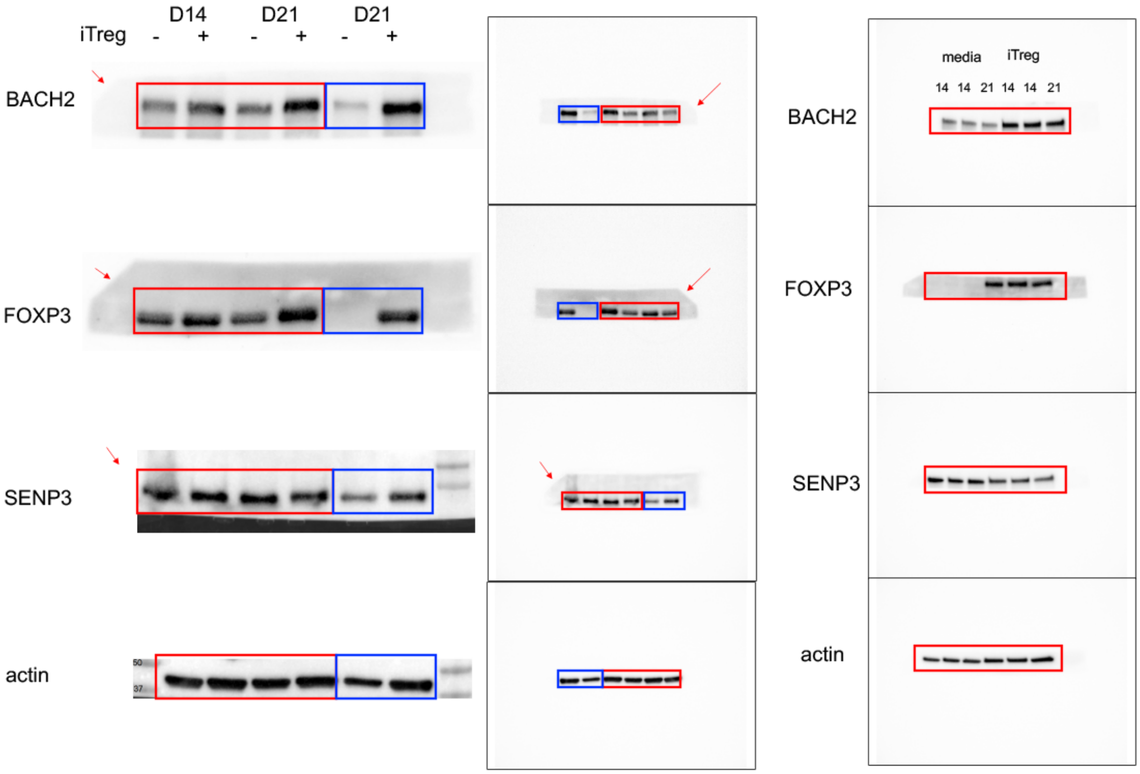

Fig. 6b

repeated data for statical analysis

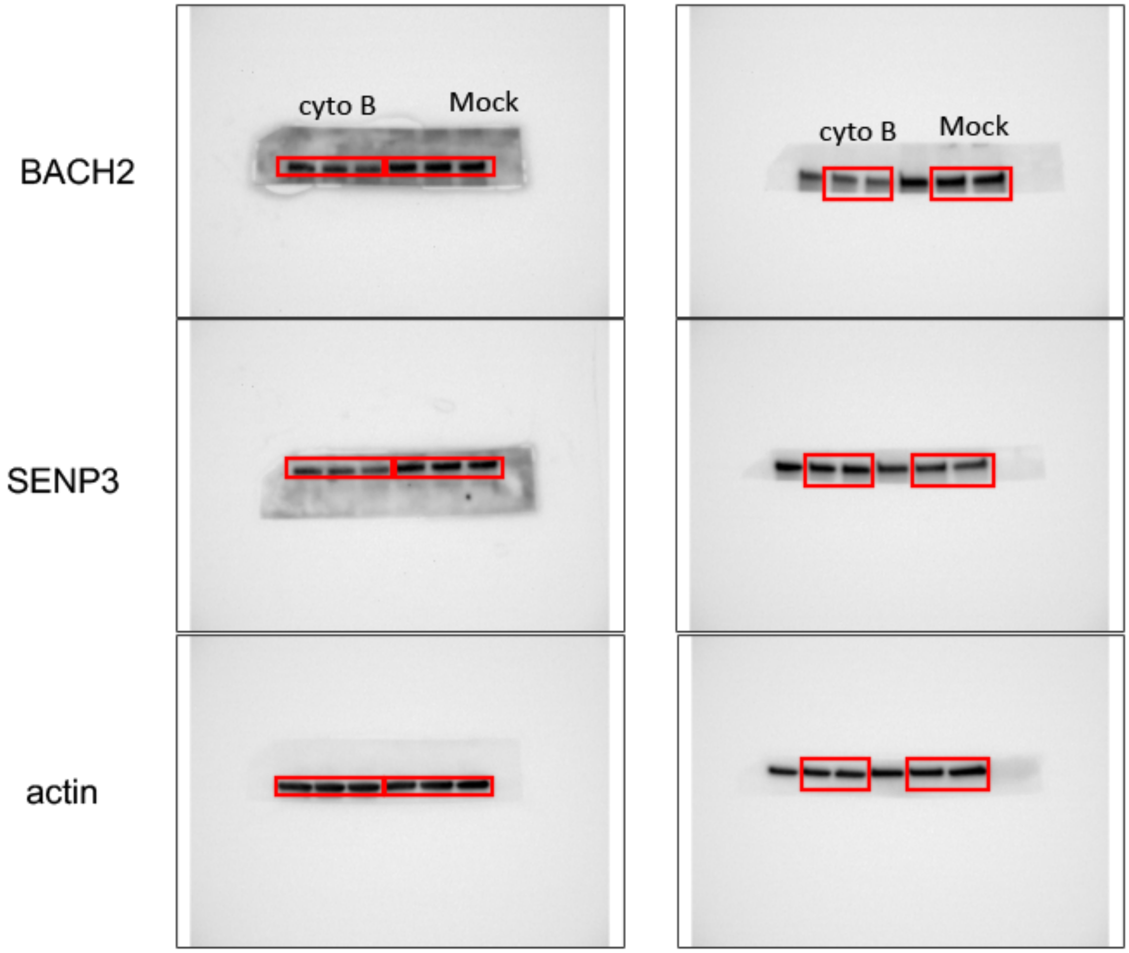

Supplemental Figures Fig. 7

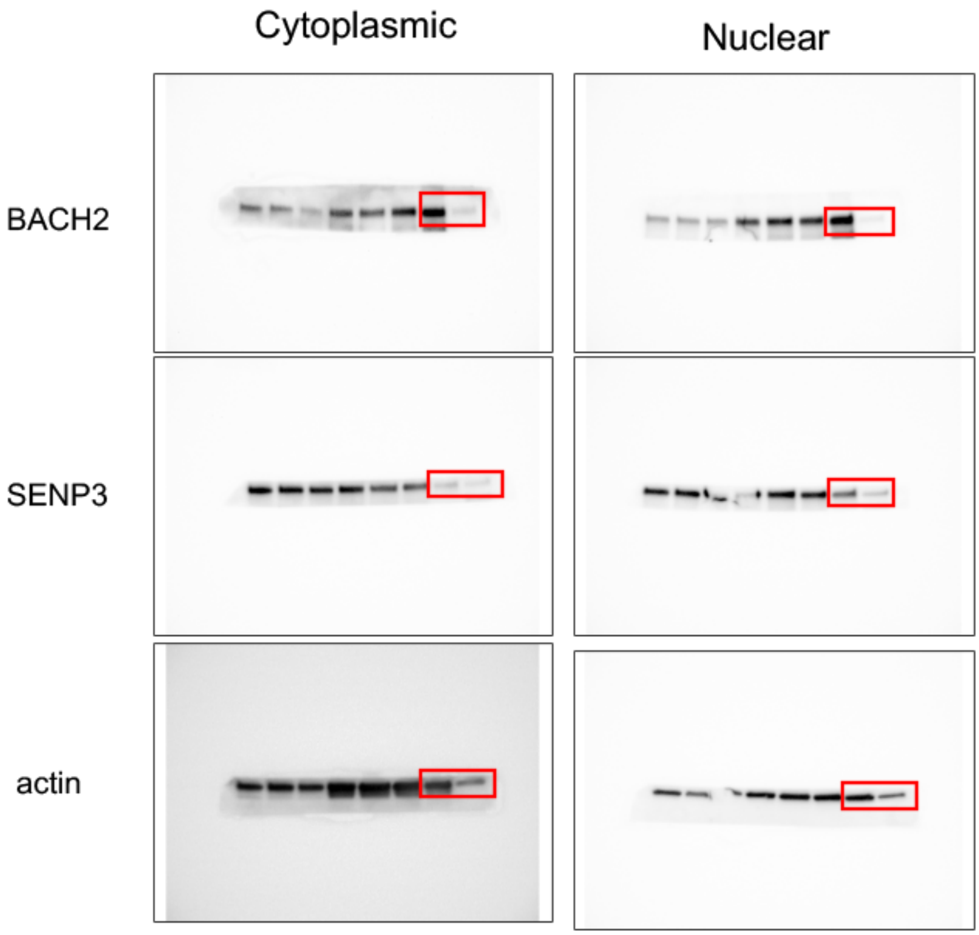

Supplement: Supplementary file 1 — Supplementary Information. [file 41598_2021_90115_MOESM1_ESM.pdf]
